# Exosome Mediated Cytosolic Cisplatin Delivery Through Clathrin-Independent Endocytosis and Enhanced Anti-cancer Effect *via* Avoiding Endosome Trapping in Cisplatin-Resistant Ovarian Cancer

**DOI:** 10.3389/fmed.2022.810761

**Published:** 2022-05-03

**Authors:** Guannan Zhou, Yuanyuan Gu, Zhongyi Zhu, Hongdao Zhang, Wei Liu, Beiying Xu, Fangyue Zhou, Menglei Zhang, Keqin Hua, Ligang Wu, Jingxin Ding

**Affiliations:** ^1^Department of Gynecology, The Obstetrics and Gynecology Hospital of Fudan University, Shanghai, China; ^2^Shanghai Key Laboratory of Female Reproductive Endocrine Related Diseases, Shanghai, China; ^3^Changning Maternity and Infant Health Hospital, East China Normal University, Shanghai, China; ^4^State Key Laboratory of Molecular Biology, CAS Center for Excellence in Molecular Cell Science, Shanghai Institute of Biochemistry and Cell Biology, Chinese Academy of Sciences, Shanghai, China; ^5^Shanghai Institute of Biochemistry and Cell Biology, University of Chinese Academy of Sciences, Shanghai, China

**Keywords:** exosome, endocytosis, cisplatin-resistant, endosome trapping, trafficking

## Abstract

**Background:**

Ovarian carcinoma is one of the most common gynecologic malignancies, cisplatin resistance has become a key obstacle to the successful treatment of ovarian cancer because ovarian carcinomas are liable to drug resistance. To find an effective drug carrier is an urgent need.

**Methods:**

Exosomes and loading-cisplatin exosomes are isolated using differential centrifugation and characterized by transmission, electron, nanoparticle tracking analysis. The anti-cancer effect of cisplatin was detected under the circumstance of delivered by exosomes or without exosomes *in vitro* and *in vivo*. Using proteome analysis and bioinformatics analysis, we further discovered the pathways in exosomes delivery process. We employed a con-focal immunofluorescence analysis, to evaluate the effects of milk-exosomes deliver the cisplatin via avoiding endosomal trapping.

**Results:**

Exosomes and exosome-cisplatin were characterized including size, typical markers including CD63, Alix and Tsg101. The anti-cancer effect of cisplatin was enhanced when delivered by exosome *in vitro* and *in vivo*. Mechanistic studies shown that exosomes deliver cisplatin mostly via clathrin-independent endocytosis pathway. Exosomes deliver cisplatin into cisplatin-resistant cancer cells clathrin-independent endocytosis and enhance the anti-cancer effect through avoiding endosome trapping.

**Conclusion:**

Cisplatin could be delivered by exosome through clathrin-independent endocytosis, and could evade the endosome trapping, diffused in the cytosol evenly. Our study clarifies the mechanism of exosomes mediated drug delivery against resistant cancer, indicates that exosomes can be a potential nano-carrier for cisplatin against cisplatin resistant ovarian cancer, which validates and enriches the theory of intracellular exosome trafficking.

## Introduction

Extracellular vesicles (EVs) are spherical bilayer small membranous vesicles generated by almost all cell types of mammalian organism. Nature derived vesicles could potentially overcome some the limitations of traditional drug delivery. Exosome (1) is a kind of the extracellular vesicles with a diameter between 30 and 200 nm (2). As a kind of emerging natural extracellular vesicles with a double-layer membrane property, exosomes possess many intrinsic strengths such as well-tolerated in the body, longer circulating half-life, able to be internalized by other cells. Another advantage (3) is that exosomes could deliver drugs across the physiological barriers including blood–brain barrier (4), one of the most intractable problem among drug delivery system. What's more, exosomes are available to be modified for better targetability (5, 6). Some studies (7) had reported that exosomes could act as nano-carriers in transporting the anti-cancer drugs such as PTX and DOX *in vitro* and *in vivo*. In addition, exosomes were reported to deliver the paclitaxel to multiple drug resistance cancer cells (8) which overexpressed P-glycoprotein, a kind of protein responsible for the drug resistance. Indeed, it has been demonstrated (4) that exosomes can serve as carriers for direct delivery of their payload chemotherapeutic drugs into cells which enables the full functionality of the chemotherapeutic drugs. These above characterizations show that exosomes [including milk exosomes (9, 10)] may play a potential role as nanocarriers for the delivery of therapeutics in drug-resistant tumors (11). In our recent study (12), we isolated exosomes from stem cells and successfully applied them in efficient cell proliferation and relevant regulation. The same method is applied to isolate exosomes which derived from diverse original resources.

Ovarian carcinoma is one of the most common gynecologic malignancies in the world (13, 14), and the associated mortality rate is the fifth highest among gynecologic malignancies. Cisplatin and its derivatives carboplatin are platinum-based chemotherapeutics (15) widely used as first choice in ovarian carcinoma (16, 17), but cancer cells are liable to drug resistance (18, 19). Thus, the application of platinum-based chemotherapeutics is restricted once platinum resistance occurs. As the result of drug resistance and further treatment dilemma, the 5-year survival rate of ovarian cancer patients is not satisfied, only <30% (20, 21). Cisplatin resistance has become a key obstacle to the successful treatment of ovarian cancer.

There are two main mechanisms of cisplatin resistance: one mechanism is cisplatin trafficking, which is mainly dependent on the major plasma membrane transporter named hCtr1 (22–25), which engaged in the uptake of cisplatin. Cisplatin cannot diffuse freely across lipid membranes due to the hydrophilic nature, and its transport across the plasma membrane and organelle membranes is mediated by hCtr1, thus also be restricted by the carrier protein hCtr1. Another main mechanism is endosome trapping (22), also known as endosomal sequestration (26), was shown to influence the subcellular distribution of cisplatin. Since the target site of cisplatin is intranuclear DNA, cisplatin trapped in the endosome will not have access to their targets. Therefore, endosomal trapping including lysosomal compartmentalization of cisplatin would lower the drug concentration at the target sites, thereby hindering their ability to exert a cytotoxic effect in ovarian cancer cells. Above all, it is crucial to investigate a kind of nanocarrier to overcome drug resistance while delivering the chemotherapeutics into drug-resistant cancers.

In this study, we firstly founded that the milk-derived exosome can sever as a potential drug nanocarrier to deliver cisplatin to inhibit the cisplatin-resistant ovarian carcinoma *in vitro* and *in vivo*. However, the mechanism of overcoming drug resistance has not been elucidated. Then, we demonstrated that milk-exosome drug delivered cisplatin through endocytosis process and further evade endosome trapping to against the endosome obstacle in cisplatin resistant ovarian cancer cells, which explains the results of enhanced anti-cancer efficacy both *in vitro* and *in vivo*. We envision that the high potency of milk-exosomes in cytosolic cis-platinum delivery might renew the interest in using drug delivery system in human cancer therapy. Understanding the endocytosis process and evading endosomal trapping mechanism allowed us to illustrate the vesicle trafficking in cancer cells and further develop the upgraded exosomes with optimal drug delivery efficiency, thereby efficiently overcome the drug resistance in cancer therapy.

## Materials and Methods

### Cell Culture

Human ovarian cancer cell line A2780CP cells were endowed generously from the obstetrics and gynecology hospital of Fudan University, and A2780CP cells were cultured in Dulbecco's Modified Eagle Medium (DMEM; Thermo Fisher Scientific Life Sciences, Massachusetts, USA) containing 10% v/v fetal bovine serum (FBS) and 100 U/mL penicillin & streptomycin. Cells were cultivated in T25 flask (Corning, NY, USA) at 37°C and 5% CO2 under humidified conditions and reached 70% density before use.

### Materials

Raw milk treated using membrane filtration sterilization rather than pasteurization was purchased (Shanghai Bright Diary Group Co., Ltd., Shanghai, CN) for exosome isolation. Cytochalasin D, chlorpromazine, simvastatin, genistein and EIPA were purchased form Sigma-Aldrich. Before treated with milk-exosome/cis or simple cisplatin, A2780 cells were pretreated with inhibitors including cytochalasin D (10 uM), chlorpromazine (25 uM), genistein (8 ug/mL) and EIPA (100 uM) for 30 min. For the pretreatment of simvastatin (2 uM), cells were pretreated for 20 h. all the above inhibitors were present throughout the experiment. FITC-conjugated cisplatin (cis-FITC) (Xi'an ruixi Biological Technology Co., Ltd., Xi'an, CN) was purchased for the subsequent encapsulation for milk-exosome/cis-FITC. Cisplatin (CSN pharm, Chicago, USA) was purchased for the subsequent study. Copper sulfate were purchased from Sigma-Aldrich.

### Isolation of Exosomes and Their Characterization

Exosomes were isolated from mature bovine milk by differential centrifugation as described preciously according to Thery's protocol (27). The isolated exosomes were characterized by the analysis of size and surface protein markers (CD63 and CD81). The protein markers were characterized by western blotting as described. The exosomes were suspended in PBS and stored at −80°C until use. The size distribution of the isolated exosomes was measured by using a NanoSight NS300 (Malvern, Amesbury, GB). The morphology and size were observed by transmission electron microscopy (FEI Tecnai G2 Spirit Twin, Philips, NL) as described previously (12).

### Preparation and Quantification of Milk-Exosomes Loaded Cisplatin

The drug loading of cisplatin (milk-exosomes/cis) was conducted by mixing of cisplatin solution (acetonitrile: ethanol in 1:1 v/v) with the milk-exosome dispersion in the proportion of 1:9 at room temperature, as previous described (28). Unbound cisplatin was removed by centrifugation at 10,000 g for 30 min, and the milk-exosomes/cis were isolated by centrifugation at 135,000 g for 2 h. The pellet was suspended in PBS and stored at −80°C. Drug loading was determined by analysis of ultra-performance liquid chromatography (Shimadzu, Kyoto, Japan). Briefly, 0.95 ml acetonitrile was added to 50 μL of milk-exosomes/cis to extract the cisplatin and precipitate the milk-exosomal proteins. The precipitated proteins were separated by centrifugation at 10,000 g for 10 min, supernatant was separated and 5 μL samples were analyzed on UPLC system using a Shim-Pack XR-ODS II reverse phase column (Shimadzu; 150 × 3.0 mm i.d., 2.2 μm). Acetonitrile and water were used to form the standard curves. The cisplatin was detected at 254 nm and total cisplatin concentration was calculated against the standard curves of cisplatin.

### Protein Determination

To quantify exosomes, the protein content was measured by Bradford assay kit (New Cell & Molecular Biotech, Suzhou, CN). Exosome preparations, usually diluted by 10-fold, were compared in triplicates against serially diluted BSA as standard according to manufactures instructions. Values were extrapolated from this curve, using a three-order polynominal equation, with R^2^ > 0.99 for each assay.

### Western Blot Analysis

Cells or exosomes were harvested and lysed in RIPA buffer (50 mM Tris-HCl, pH 7.4, 150 mM NaCl, 1% Triton X-100, 1% Na deoxycholate, 0.1% SDS, 1 mMEDTA) supplemented with complete protease inhibitor cocktail (Sigma-Aldrich). Lysates were centrifuged at 12,000 g for 30 min at 4°C to remove insoluble material, after which protein concentrations were determined using a Bradford assay kit using bovine serum albumin (BSA) as a standard according to the manufacturer's instructions. Proteins were resolved on SDS-polyacrylamide gels, and then transferred to a polyvinylidene difluoride (PVDF) membrane. After blocking with 5% (w/v) fat free milk, the membrane was stained with the corresponding primary antibodies followed by incubation with the appropriate secondary HRP-conjugated antibodies. The membrane was incubated with the following primary antibodies: anti-CD63 antibody (Abcam, ab134045), anti-TSG101 antibody (Abcam, ab125011), anti-ALIX antibody (Abcam, ab88388) and anti-CAV1 antibody (Abcam, ab2910), anti-ARF6 antibody (Abcam, ab131261), anti-Rac1 antibody (Abcam, ab155938), anti-CLTC antibody (Abcam, ab21679), anti-GAPDH (Cell signaling).

### Exosome Uptake Analysis

Confocal microscopy was performed for detection of the uptake of exosomes, milk-exosome/cis were stained with PKH26 Dye (PKH26 Red Fluorescent Cell Linker Kits, Sigma-Aldrich, Merck KGaA, Darmstadt, GER) to evaluate the internalization by A2780CP cells. Milk-exosomes diluted in 1.5 mL Diluent C (PKH26 Red Fluorescent Cell Linker Kits, Sigma-Aldrich, Merck KGaA, Darmstadt, GER), then were mixed with the mixture containing 6 μL PKH26 dye and 1.5 mL Diluent C following the protocol of the manufacturer. After 10 min, 6 mL ultracentrifuged exosome-free-FBS was added and incubated for 5 min to bind the excess dye. Then 15 mL DMEM were added to wash the product, followed ultracentrifugation again at 1,25,000 g for 60 min at 4°C. The supernatant was removed as completely as possible and purified PKH26-labeled-milk-exosome/cis were resuspended with 1 mL DMEM. Then, 1 mL of 5,000-ng/mL PKH26-labeled-milk-exosome/cis in DMEM were incubated with A2780CP cells/3 × 104 per well in 24-well-plate containing round coverslips at 37°C under 5% CO2 condition. Anti-fluorescence-quenching agent with DAPI (6-diamidino-2-phenylindole) (DAPI Fluoromount-GTM; Yeasen Biotechnology, Shanghai, China) was used to mount the slides and label nuclei. Images were captured with a TCS SP5 confocal laser scanning microscopy (Leica Microsystems, Wetzlar, GER).

### Confocal Immunofluorescence Microscopy

The localization of exosomes, cargos, and other organelles in cultured cells, as described before. Milk-exosome/cis-FITC was prepared according to the protocols above, by using milk-exosome encapsulate the FITC-labeled-cisplatin (cis-FITC). A2780CP cells were seeded in 24-well-plate containing round coverslips (3 × 10^4^ per well) for 12h, and pretreated with cytochalasin D (10 uM), chlorpromazine (25 uM), genistein (8 ug/mL) and EIPA (100 uM) for 30 min, then treated with milk-exosome/cis-FITC or cis-FITC. After washes three times with PBS to remove non-internalized milk-exosome/cis-FITC or cis-FITC, cells were fixed with 4% paraformaldehyde (PFA) in PBS for 10 min at 25°C, then washed three times with Immunol Staining Wash Buffer (Beyotime Biotechnology, Shanghai, CN) containing Triton X-100, followed blocking of the cells. Endosome related organelles were stained with anti-Rab5 (Abcam, ab109534), followed by Alexa Fluor 594-conjuated secondary antibodies. Lysosomes were stained with LysoTracker Red (Beyotime Biotechnology, Shanghai, CN) and pretreated for 1h at 37°C before the cellular fixation. After that, cells were washed again with Immunol Staining Wash Buffer for three times. Anti-fluorescence-quenching agent with DAPI (6-diamidino-2-phenylindole) (DAPI Fluoromount-GTM; Yeasen Biotechnology, Shanghai, China) was used to mount the slides and label nuclei. Images were captured with a TCS SP5 confocal laser scanning microscopy (Leica Microsystems, Wetzlar, GER). The fluorescence was quantified using ImageJ software (NIH, USA).

### Antiproliferative Activity *in vitro*

A2780 cells were seeded in a 96-well-plate (10^4^ cells per well) and cultured for 12–24 h, subsequently they were treated with cisplatin (5 μg/mL), milk-exosome/cis [cisplatin (5 μg exosomal cisplatin/mL)], milk-exosome, and PBS as control for 24 h and the cell viability was measured by a tetrazolium dye reduction assay called CCK-8 (Dojindo, Japan). Optical density (OD) value was measured by spectrophotometric measurement at 450 nm and calculated after removing the mean background values. The milk-exosome/cis concentration measurement is depend on the cisplatin in this study.

### Antitumor Experiment in Animal Model

The animal experiments were conducted in accordance with the criteria for the Care and Use of Laboratory Animals and approved by the Ethics Committee of Obstetrics and Gynecologic hospital of Fudan University. Female athymic nude (nu/nu) mice (5–6 weeks old) were purchased from Jiesijie Laboratory Animal Co., Ltd., Shanghai, China. And maintained according to the Institutional Animal Care and Use Committee guidelines. Ovarian tumor xenografts were produced by subcutaneously injecting human ovarian A2780CP cancer cells (2.5 × 10^6^) in serum-free media in the left flank of the mice. Mixture of cisplatin (4 mg/kg b.wt.) with milk-exosomes, and milk-exosome/cis (4 mg exosomal cisplatin/kg b.wt.) were injected intravenously (i.v.) into the nude mice every 3 days after the tumoral diameters had increased to 5 mm, and the sizes of the tumors were measured every 3 days. After 21 days, the mice were killed, body weight and tumor volume were measured weekly until termination of the study. The animals were euthanized by CO_2_ asphyxiation when the tumor volume reached ~800 mm^3^.

Luciferase-labeled A2780CP (2 × 10^5^ cells) were injected into the 4-week-old female BALB/c-nu mice. Immediately after implantation, mice were injected with mixture of cisplatin (4 mg/kg b.wt.) with milk-exosomes, and milk-exosome/cis (4 mg exosomal cisplatin/kg b.wt.) intravenously, every 3 days. Bioluminescence imaging was carried out using a Xenogen system (IVIS spectrum, Perkin Elmer, USA).

### Transfection Assays

A2780 cells were seeded at a density of 50,000 cells/well in 6-well-plates. After 24 h, cells were transfected with siRNA (sequences are listed in Supplementary Table S1) using Lipofectamine RNAiMax (Life Technologies) according to the protocols. Repeat the transfection 24 h later, replaced the medium at 6 h after the second transfection. From then on, cells be incubated for another 24 h for milk-exosome/cis internalization assay, or cells be incubation 48 h for Western blot evaluation.

### Proteomic Analysis of Milk Exosomes

For proteomic analysis, lysis buffer was added into the collected sample and cracked using the ultrasonic cell breaker for 1 min. The lysate was then to be centrifuged at 13,000 rpm for 20 min at 4°C. Collected the supernatant, and acetone was added to a 6:1 ratio (acetone to supernatant). Incubated the mixture at −20°C overnight, then centrifuged to collect the protein pellet. Washed the pellet and redissolved it in buffer (300 mM triethylamine borane, TEAB, 6 M guanidine hydrochloride), followed by centrifuging 13000 rpm for 10 min at 4°C. Then, quantitated the protein by the BCA assay. Digested protein was performed according to the filter-aided sample preparation (FASP) procedure. Briefly, after protein solution samples were taken from each sample, the volume was determined to 100 μL with 25 mM ammonium bicarbonate. Then, 1 M DTT was added (terminal concentration 20 mM), and the reduction reaction was kept for 1 h at 57°C. Subsequently iodoacetamide was added (terminal concentration 90 mM) and incubated for 40 min at room temperature under dark conditions. The sample solution was centrifuged on a 10 kDa ultrafiltration tube at 12000 rpm, and ammonium bicarbonate was added into the ultrafiltration tube to wash 4 times. The sample was digested with trypsin which was diluted with ammonium bicarbonate at 37°C overnight. Next day the peptides were collected after centrifugation, dried by centrifugal concentration. Then, collected the digested peptides, concentrated and dried by centrifugation. Desalted the peptide of each sample using the Monospin C18 column. After drying, they were prepared for machine analysis of mass spectrometry (Orbitrap Fusion Lumos, Thermo Scientific, USA). The dried sample was dissolved in buffer (0.1% FA), analyzed by mass spectrometry. The on-line Nano-RPLC liquid chromatography was performed by Easy-nLC 1200 system (Thermo Scientific). The trap column was home-made C18 (C18, 5 *u*m, 100 *u*m^*^2 cm) and the analytical column was C18 reversed-phase column (C18, 1.9 μm, 75 μm × 200 mm), Peptides were eluted from the trap column at 200 nl/min with an increasing concentration of Buffer (0.1% formic acid in acetonitrile) over a 120 min gradient. The peptides results were subjected to nano electrospray ionization source followed by tandem mass spectrometry in Orbitrap Fusion Lumos (Thermo Fisher Scientific). The mass spectrometer was operated in the data-dependent mode. For MS scans, the scan ranged from 375 to 1,600 m/z. Intact peptides were detected at a resolution of 60,000. Collision energy: 30% HCD. For database searching, the Proteome Discovery2.4 (Thermo, PD 2.4) was used to search against the Swiss-Prot database (www.uniprot.org. October 2019 released). The search was performed for trypsin-digested peptides with 2 allowed missed cleavages permitted. Fixed modification of carbamido-methylation and variable modifications of oxidation (M) and acetylation (N-terminal) was defined. Peptide counts and peptide intensities are used to calculate total protein Normalized Abundances for each protein group. For the study of the biological functions of identified proteins, and to reveal which pathways were significantly represented by the identified proteins, the Gene Ontology (GO) analysis and the pathway analysis were performed using the database of DAVID 6.8.

### Statistical Analysis

SPSS 25.0 (IBM Corporation, Armonk, NY) and GraphPad Prism (GraphPad Software, Inc., San Diego, CA, USA) software were used for statistical analyses. Each value is expressed as the mean and standard error of the mean (SEM) of multiple determinations. Statistical analyses were performed with Student's *t*-test for comparisons between two groups and with ANOVA followed by Dunnett's correction for more than two groups. *P*-value of 0.05 or less was considered as statistically significant.

## Results

### Preparation and Characterization of Milk-Exosome/Cisplatin

**Figure 1:**
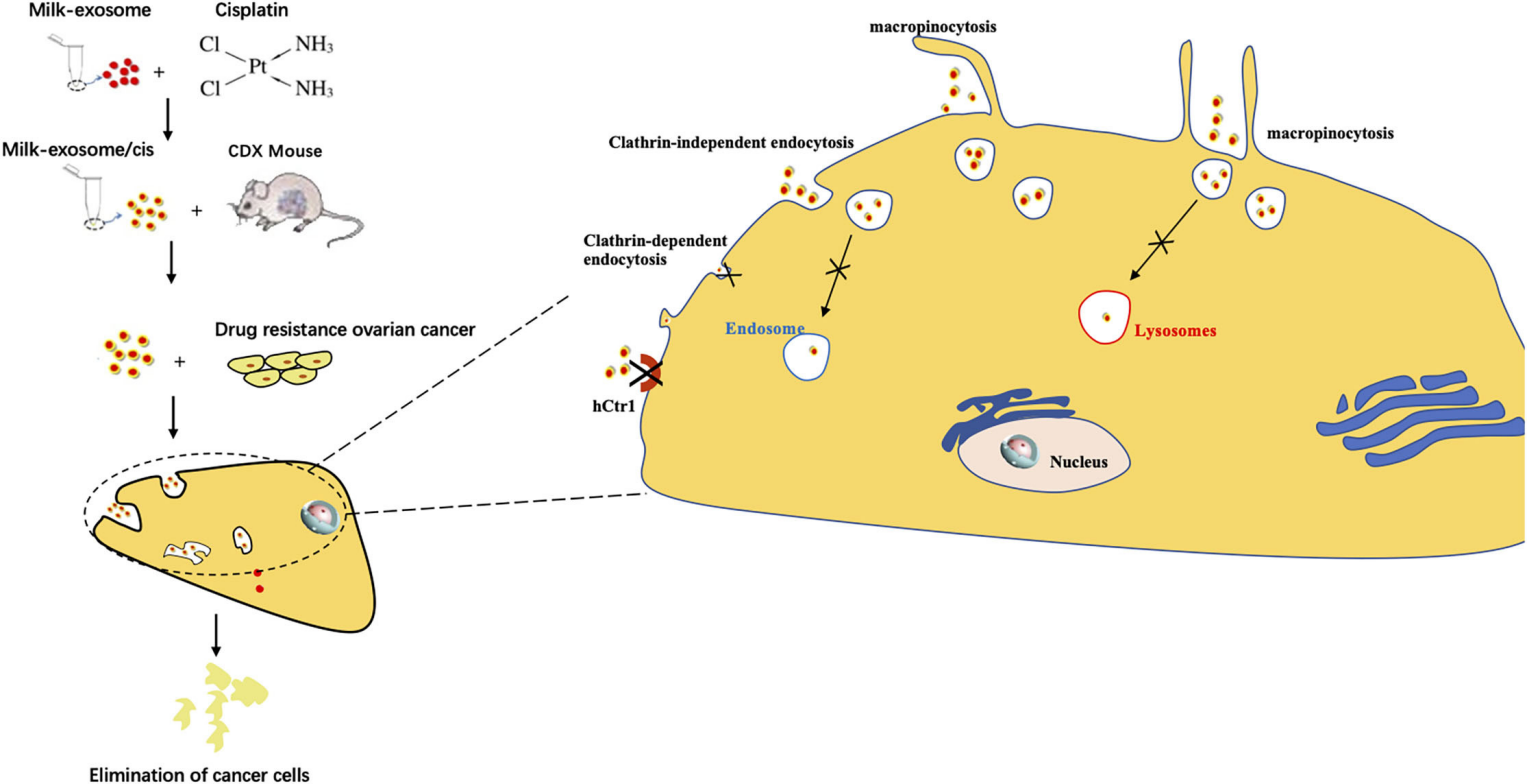
Graphical abstract of this study. Exosome mediated cytosolic cisplatin delivery and enhanced anti-cancer effect in cisplatin-resistant ovarian cancer *via* avoiding endosome trapping.

**Figure 2:**
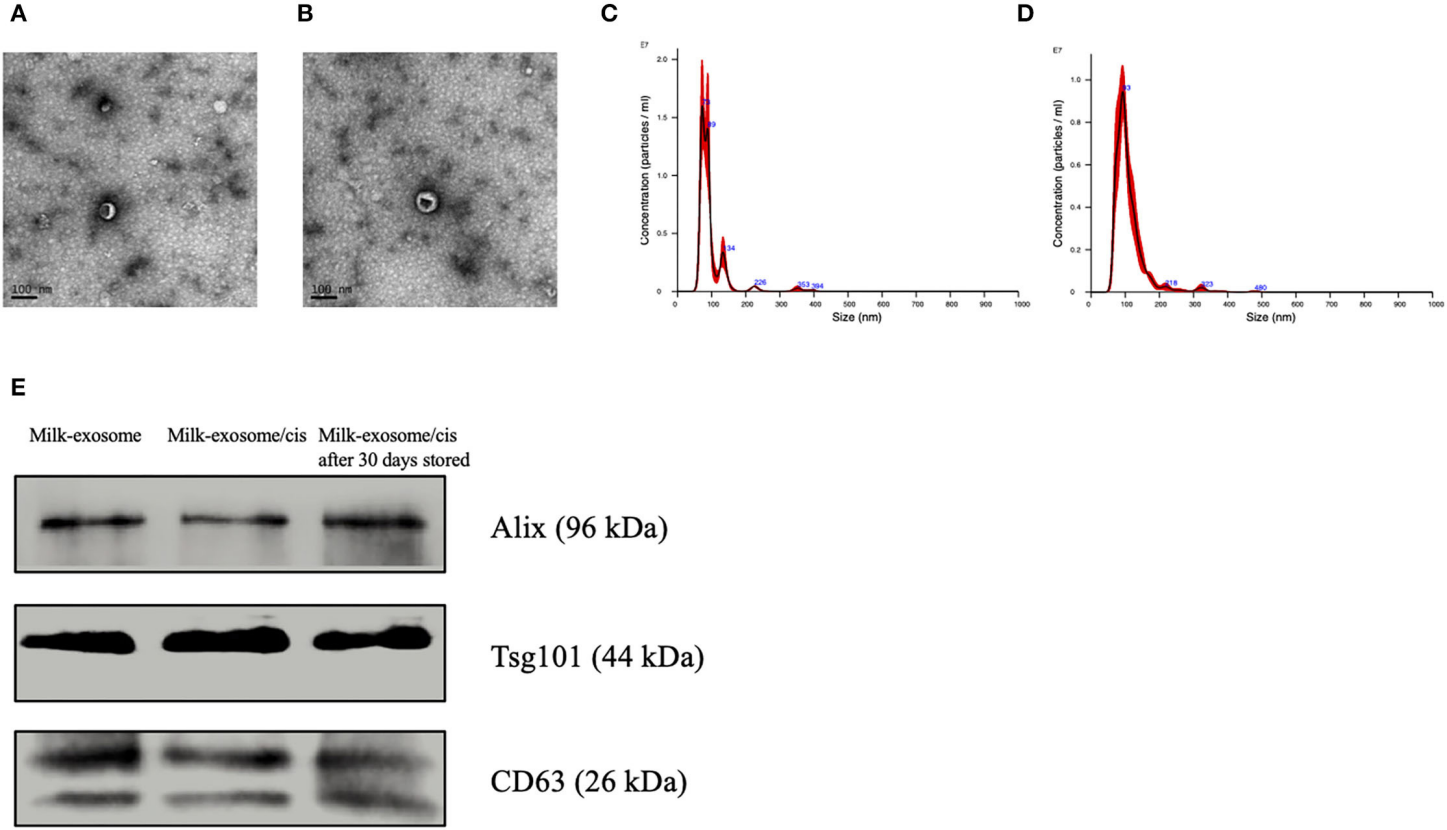
Characterization of milk-exosomes and milk-exosome/cis. **(A,B)** Electron microscopy analysis of milk-exosomes and milk-exosome/cis. Scale bar represents 100 nm. **(C,D)** Size distribution profile of milk-exosomes and milk-exosome/cis as detected by NanoSight. **(E)** Western Blot analysis of Alix, CD63 and Tsg101 in milk-exosomes, milk-exosomes/cis and stored milk-exosomes/cis.

The scheme to prepare milk-exosome-loaded-cisplatin (milk-exosome/cis) is presented in Figure 1. We isolated milk exosomes from fresh raw milk by differential centrifugation according to the methods of Thery's protocol (27). Critical quality attributes of milk-exosome and milk-exosome-cisplatin were demonstrated (Figure 2). The milk-exosome and milk-exosome/cis were largely 30-150 nm in diameter, as measured by NanoSight (Figures 2C,D, Supplementary Table S2), the diameter of milk-exosome/cis is larger than that of milk-exosome. The cup shape of milk-exosome and milk-exosome/cis were observed by transmission electron microscope (Figures 2A,B), revealed that the cup shape of exosome nanoparticles remained unchanged after cisplatin loading in milk-exosome/cisplatin formulation. Also, western blot showed traditional exosomal markers including Alix and CD63, were expressed in isolated milk-exosome and milk-exosome/cis (Figure 2E, Supplementary Figure S1).

### The Cisplatin Loading Ability of Milk-Exosome

The loading ability of milk-exosomes was assessed after solvent extraction of the milk-exosome/cis, followed by analysis of the cisplatin by UPLC assay. According to the results that the milk-exosome/cis maintain similar size, cup shape and exosomal markers, we deduced that cisplatin was encapsulated into the milk-exosomes. As analyzed by UPLC, results suggested that the encapsulation ability of cisplatin is 18% based on the loading method in the present study (Supplementary Table S2, Supplementary Figure S2). The drug loading ability in this study is in line with the results as previous reported (28, 29).

### Enhanced Anti-cancer Effects by Milk-Exosome Drug Delivery *in vitro* and *in vivo*

**Figure 3:**
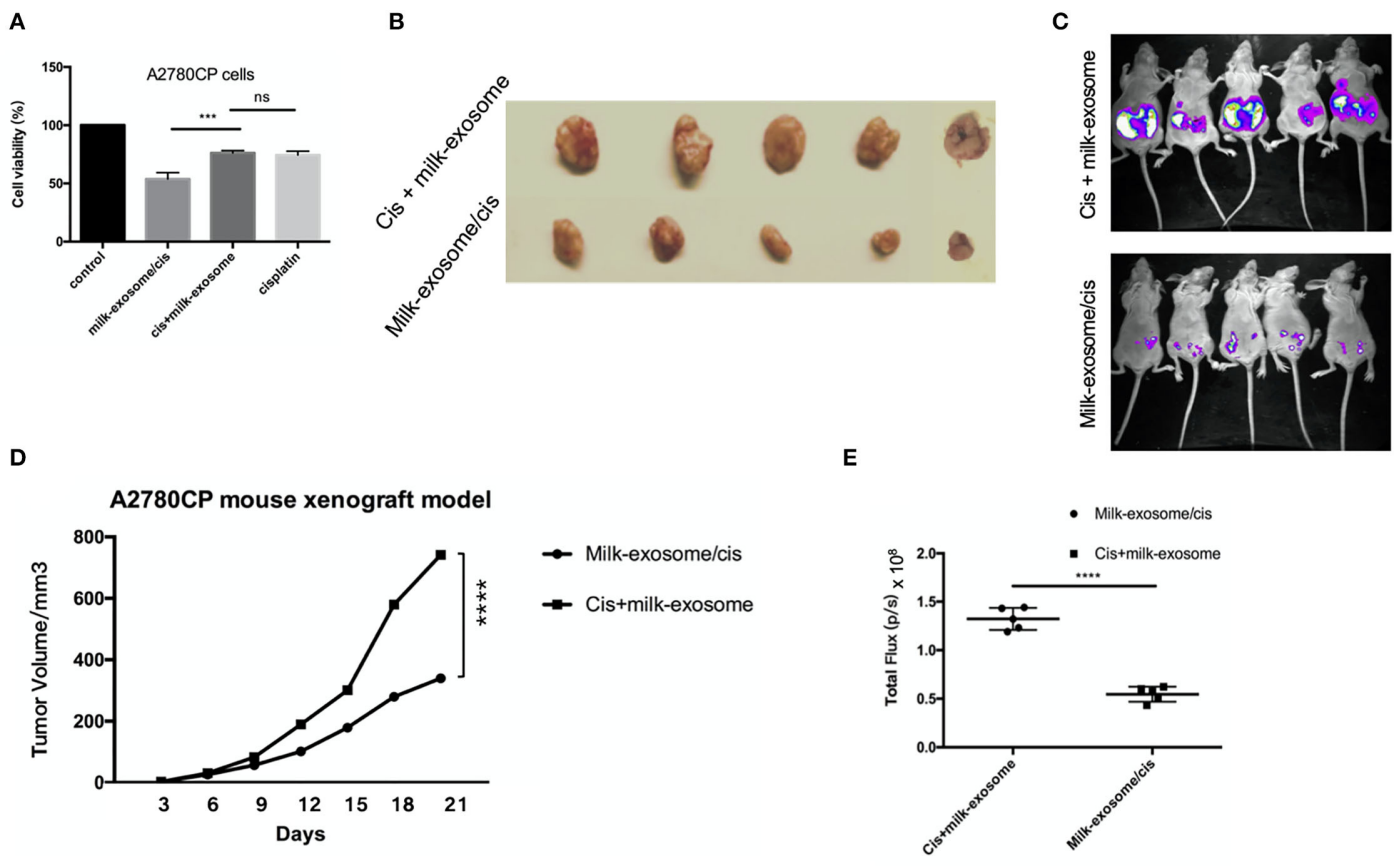
Effect of milk-exosome loaded cisplatin on cisplatin-resistant ovarian cancer *in vitro* and *in vivo*. **(A)** The cisplatin-resistant ovarian cancer A2780CP cells were treated with cisplatin (5 μg/mL), milk-exosome/cis (5 μg exosomal cisplatin/mL), milk-exosome, and PBS. CCK8 assays were conducted after 24 h. Three independent experiments with biological repeats. **(B)** Milk-exosome/cis enhanced the anti-cancer effect *in vivo*. Nude mice were subcutaneously injected with A2780CP cells. Mixture of cisplatin (4 mg /kg b.wt.) with milk-exosomes, and milk-exosome/cis (4 mg exosomal cisplatin/kg b.wt.) were injected every 3 days. Gross appearance of the tumors. **(C)** The size of the tumors was measured every 3 days. **(D)** Milk-exosome/cis enhanced the anti-cancer effect in bioluminescent ovarian cancer model *in vivo*. **(E)** Bioluminescent images were visualized and calculated. ****, *P* < 0.0001 and ***P < 0.001. ns, no significance.

One of the important evaluation criteria upon drug nanocarrier delivery is whether the delivered therapeutics enhance the anti-cancer effects when compared with simple treatment without drug nanocarrier delivery. Milk-exosomes/cis was tested to evaluate their inhibition effects on cisplatin-resistant ovarian cancer growth *in vitro* and *in vivo*. The *in vitro* anti-cancer effect of cisplatin delivering *via* milk-exosomes was evaluated in resistant ovarian cancer A2780CP cells, milk-exosome/cis was compared with the simple mixture of milk-exosome and cisplatin when added in A2780CP cells (Figure 3A). The results showed that milk-exosome/cis had a higher anti-cancer effect than the simple mixture of milk-exosome and cisplatin, suggesting that milk-exosomes could enhance the anti-cancer effects of cisplatin in cisplatin-resistant ovarian cancer cells.

Similarly, the inhibition effect of milk-exosome/cisplatin was tested in A2780CP cells derived cancer xenograft mouse model. The *in vivo* anti-cancer effect of cisplatin delivering *via* milk-exosomes was evaluated in the cisplatin-resistant ovarian cancer A2780CP cells derived cancer xenograft mouse model. Cisplatin (4 mg/kg b.wt.) and milk-exosome/cis (4 mg exosomal cisplatin/kg b.wt.) were intravenously injected every 2 days. As demonstrated (Figures 3B,D), the group treated with milk-exosome/cis significantly suppressed *in vivo* tumor growth as measured by tumor volume, compared to the control group of cis-platinum without milk-exosome. Moreover, in the bioluminescent mouse model established by cisplatin resistant luciferase-labeled A2780CP cells, milk-exosome significantly inhibited ovarian cancer when compared to the simple cisplatin mixed with milk-exosomes (Figures 3C,E).

### Internalization of Milk-Exosome/Cisplatin by A2780CP Cells

**Figure 4:**
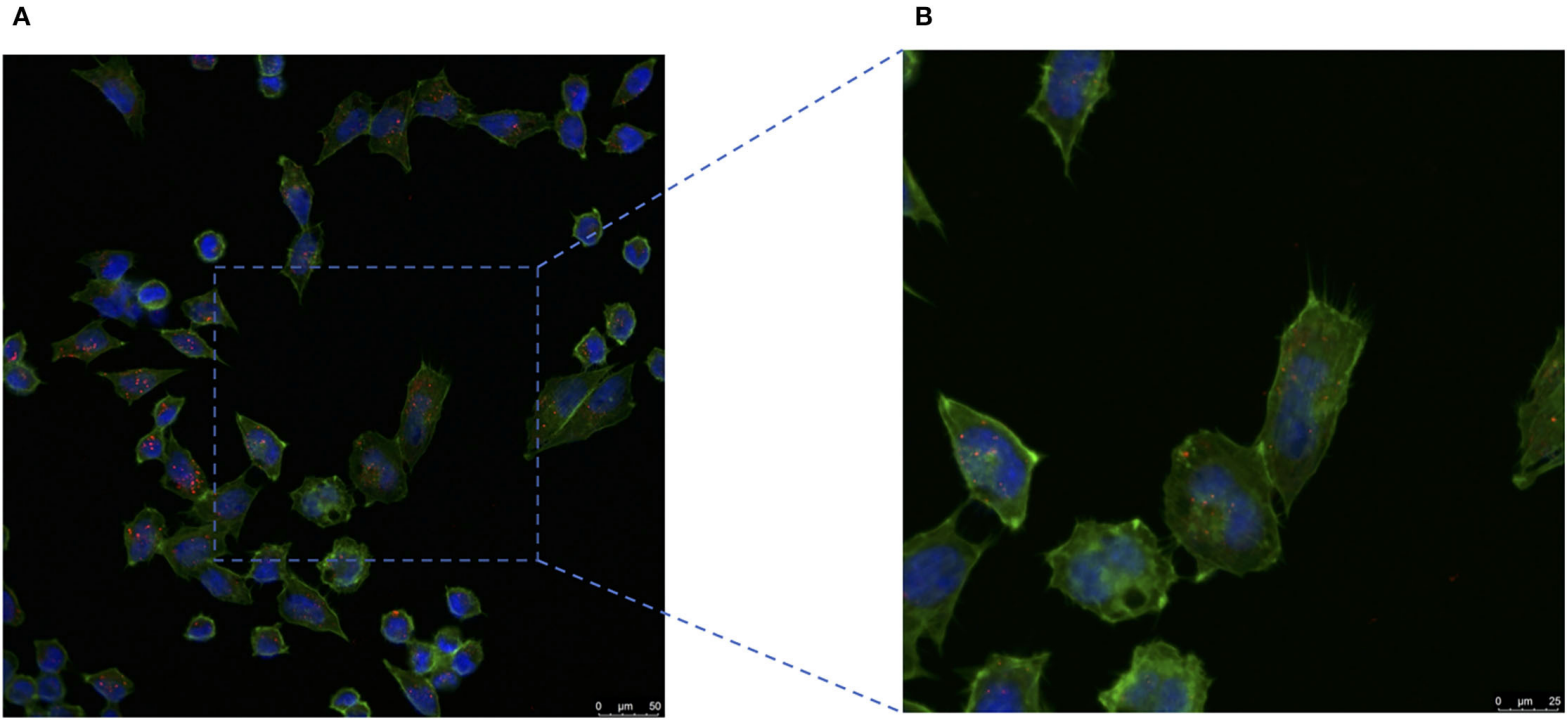
Cisplatin-resistant ovarian cancer A2780CP cells internalize pkh26 labeled milk-exosome/cis. **(A)** The presence of milk-exosome/cis in A2780CP cells was analyzed by confocal microscopy after they had been incubated with PKH26 labeled milk-exosome/cis (red). The nuclei of the A2780CP cells were stained with DAPI (blue) and their cytoskeleton with Actin-Tracker Green (green). **(B)** Higher magnification of the area marked by square. Scale bars are shown to the right.

To confirm the milk-exosome/cisplatin could be internalized by cisplatin-resistant ovarian carcinoma cell line A2780CP cells, confocal microscopy analysis was conducted. Milk-exosome/cis were labeled with PKH26 red dye, the A2780CP cells were labeled by Green actin-tracker, as previous described (12). The PKH26 labeled milk-exosome/cis distributed inside the cells (Figure 4). These observations suggested that the milk-exosomes/cis were internalized in the cisplatin-resistant ovarian carcinoma A2780CP cells. The cellular uptake mechanism for PKH26 labeled milk-exosome/cis and whether the uptake process is relevant to the cisplatin transporter CTR1, needs further evaluation in this study.

### Milk-Exosome Proteome Analysis

**Figure 5:**
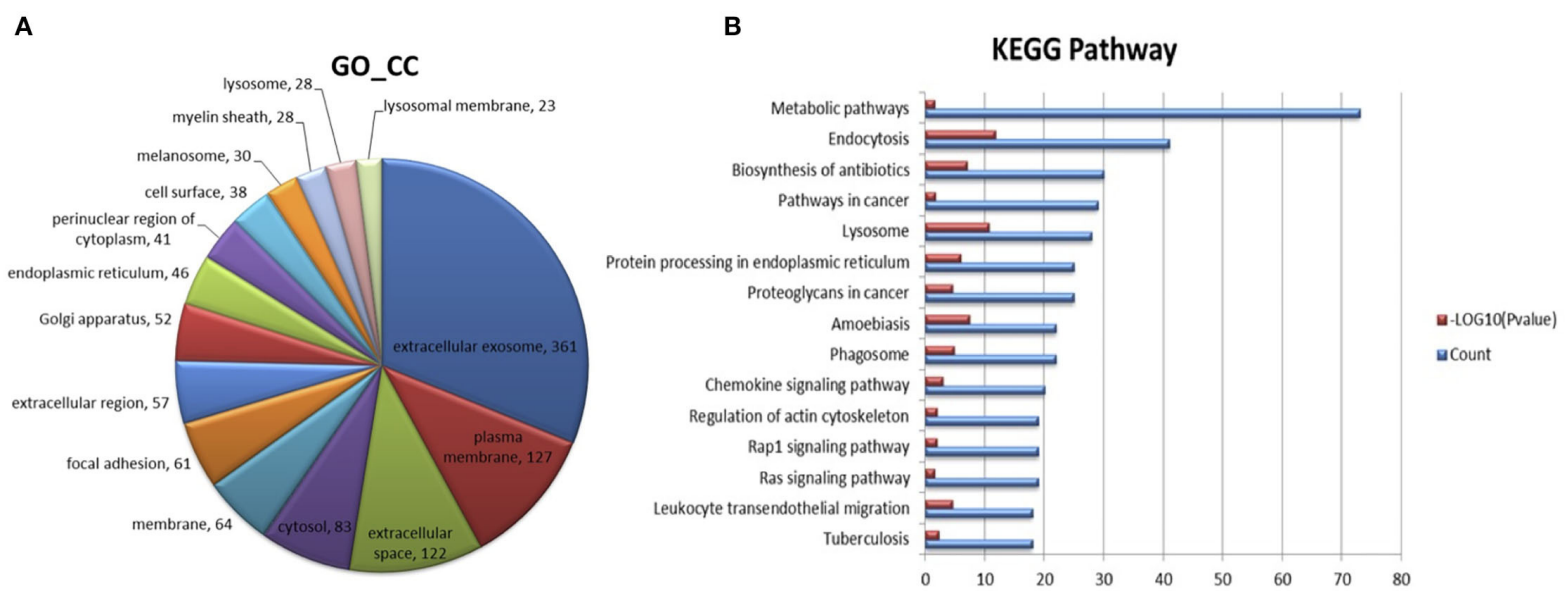
Proteome analysis of milk-exosome-enriched proteins by mass spectrometer detection and function prediction of the specifically enriched proteomes. **(A)** Abundant proteome analysis by mass spectrometer of milk-exosomes. The 15 most abundant proteomes in milk-exosomes are color labeled in the pie charts. **(B)** Functions for the proteomes significantly enriched in milk-exosomes were identified by KEGG analysis. Top 15 abundant enriched pathways are listed, endocytosis pathways involving the proteomes are shown in the list.

Figure 5A shows the GO annotations for milk exosome proteins. Not all bovine proteins have been fully annotated, therefore, of the 1,161 proteins converted into DAVID, 361 were matched to GO terms under the extracellular exosome heading, 127 were matched to GO terms under the plasma membrane heading, and 122 were matched to GO terms under the extracellular space heading. Figure 5B shows the top 15 KEGG pathways represented in milk exosome proteome by the number of milk exosome proteins identified in the KEGG pathway. Pathways (metabolic pathways, endocytosis) important in metabolism, trafficking top this limited list and are important to both milk formation and exosome transportation. This result is similarly to some previous studies (30, 31). These milk exosomes are highly enriched in endocytosis that are important in vesicle fusion and trafficking events, indicating the potential pathways during milk-exosomes trafficking.

### Endocytosis Signaling Pathway Involved in the Uptake of Milk-Exosome/cis in A2780CP Cells

To study internalization of milk-exosome/cis by A2780CP cells, milk-exosome/cis were fluorescently labeled with the membrane dye PKH67. Firstly, to figure out whether the delivery *via* milk-exosome depend on the cisplatin transporter CTR1, A2780CP cells were treated with PKH67 labeled milk-exosome/cisplatin, treated with PKH67 labeled milk-exosome/cisplatin and CTR1 inhibitor copper sulfate as control. There is no significant difference between the two groups with or without CTR1 inhibitor, while cisplatin was inhibited by CTR1 inhibitor (Supplementary Figure S3), suggesting the cisplatin delivery *via* milk-exosome is independent on the cisplatin transporter CTR1. After incubated with PKH67 labeled milk-exosome/cisplatin, the uptake process was inhibited by excessed unlabeled milk-exosome/cisplatin, also was inhibited at the temperature of 4°C, as depicted by confocal microscopy analysis (Supplementary Figure S3), which indicates that the uptake process of milk-exosome/cisplatin is mediated by active, energy-dependent specific endocytic processes rather than passive membrane fusion.

**Figure 6:**
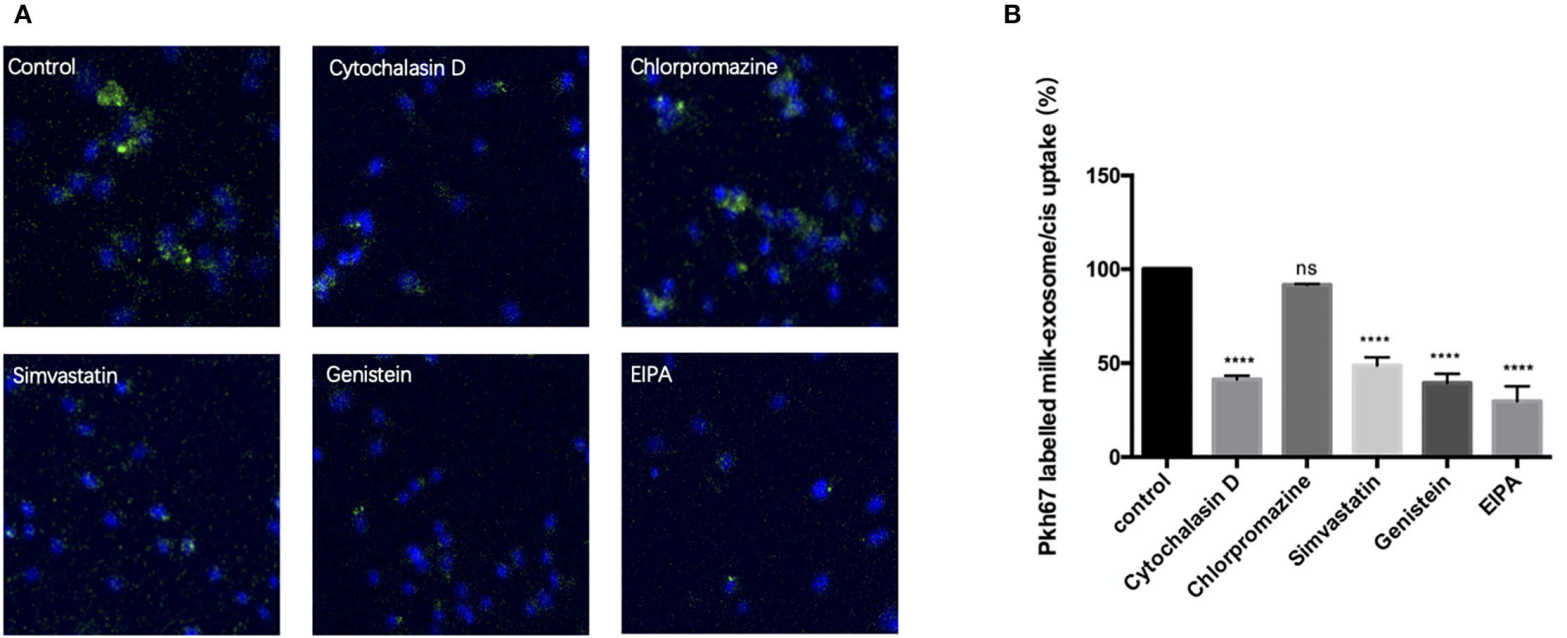
Analysis of the influence of endocytosis pathway inhibitors upon inhibiting pkh67 labeled milk-exosome/cis internalization *via* confocal microscopy. **(A)** Pkh67 labeled milk-exosome/cis uptake by A2780CP cells pretreated with endocytosis pathway inhibitors cytochalasin D (actin polymerization inhibitor), chlorpromazine (clathrin-dependent endocytosis inhibitor), genistein (clathrin-independent endocytosis inhibitor), simvastatin (cholesterol synthesis inhibitor) and EIPA (macropinocytosis inhibitor). **(B)** Quantification of pkh67 labeled milk-exosome/cis (green spots) uptake by A2780CP cells (blue fluorescence DAPI labeled nuclei) pretreated with endocytosis pathway inhibitors by confocal microscopy. Values represent mean ± S.E.M. ****, *P* < 0.0001. ns, no significance.

Furthermore, A2780CP cells were separately pretreated with cytochalasin D (actin polymerization inhibitor), chlorpromazine (clathrin-dependent endocytosis inhibitor), genistein (clathrin-independent endocytosis inhibitor), simvastatin (cholesterol synthesis inhibitor) and EIPA (macropinocytosis inhibitor), followed by incubation with PKH67 labeled milk-exosome/cisplatin. As showed in the Figure 6, the uptake process of PKH67 labeled milk-exosome/cisplatin was inhibited by cytochalasin D, genistein, simvastatin and EIPA, but was not inhibited by chlorpromazine significantly. These results suggested that milk-exosome/cisplatin were internalized mostly by clathrin-independent endocytosis and macropinocytosis, rather than clathrin-dependent endocytosis.

**Figure 7:**
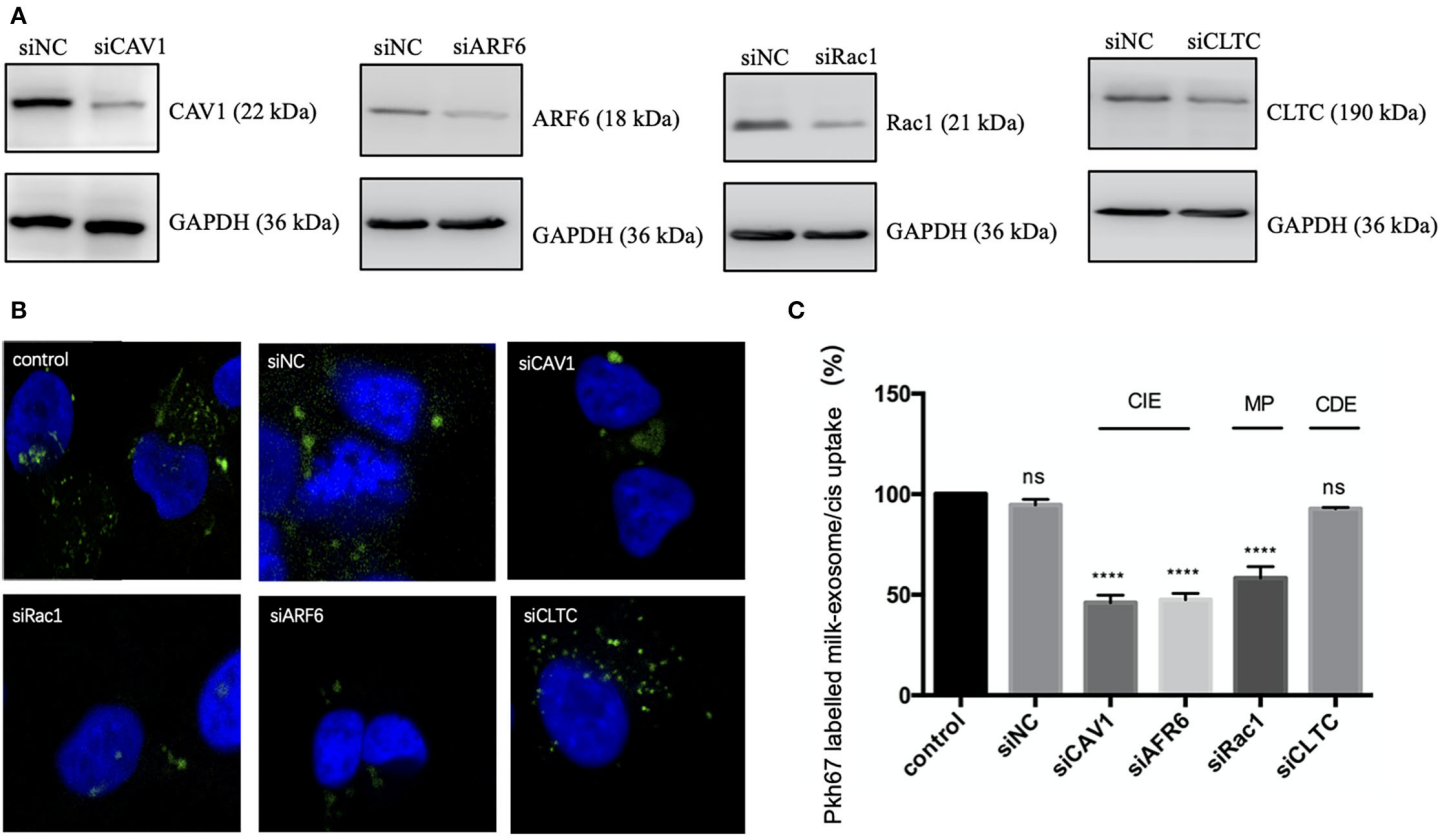
Analysis of the influence of CAV1, ARF6, Rac1 or CLTC upon inhibiting pkh67 labeled milk-exosome/cis internalization *via* knockdown technology. **(A)** Western Blot analysis of A2780CP cells transfected with siRNA against key endocytic regulators caveolin-1 (CAV1), ARF6 and Rac1 and clathrin heavy chain (CLTC), or negative control siRNA (NC). **(B,C)** Pkh67 labeled milk-exosome/cis (green spots) uptake by A2780CP cells (blue fluorescence DAPI labeled nuclei) pretreated with indicated siRNAs, as visualized and quantified by confocal microscopy **(C)**. Values represent mean ± S.E.M. ****, *P* < 0.0001. ns, no significance.

In order to verify the above observations and further figure out the key molecules involved in the endocytosis of milk-exosome/cisplatin, caveolin-1 (CAV-1), ARF6, Rac1and clathrin heavy chain (CLTC) were knockdown *via* RNAi technology (Figure 7A, Supplementary Figure S4). A2780CP cells were separately pretreated with siRNA, followed by incubation with PKH67 labeled milk-exosome/cisplatin. As demonstrated (Figures 7B,C), the uptake process of PKH67 labeled milk-exosome/cisplatin was inhibited after knockdown of clathrin-independent endocytosis regulator (CAV-1 and ARF6), macropinocytosis regulator (Rac1), but was not inhibited after knockdown of clathrin-dependent endocytosis regulator (CLTC).

### Internalized Milk-Exosome-Loaded Cisplatin Evading Endosome Trapping After Entering the Cytosol

**Figure 8:**
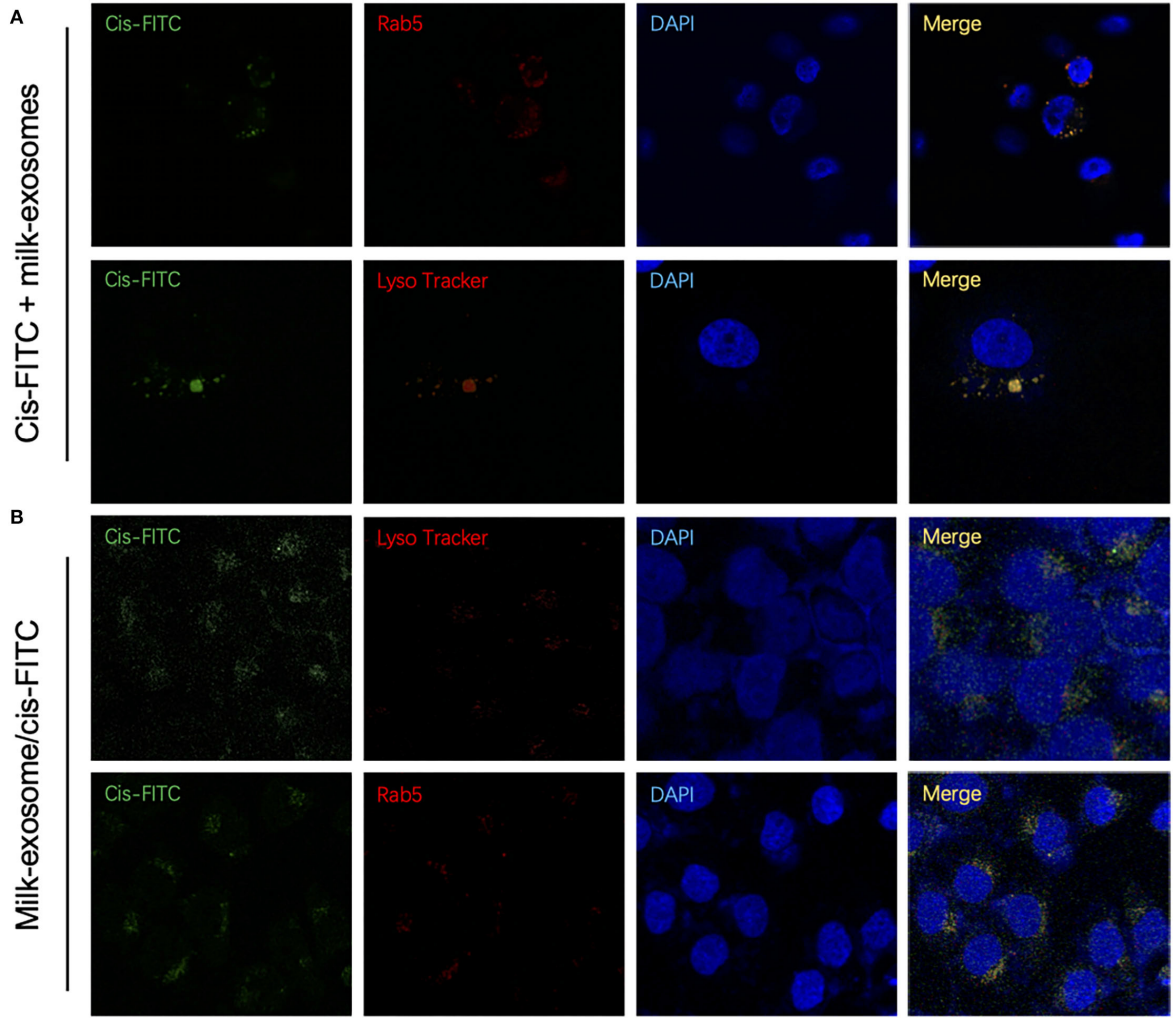
Cytosolic localization studies between cis-FITC and endosomes of cisplatin delivery with or without milk-exosomes. **(A)** Intracellular trafficking of internalized cis-FITC monitored by co-localization with the endosome marker Rab5 (red fluorescence) and lysosome marker LysoTracker Red (red fluorescence) by confocal (red fluorescence microscopy. Green represents cis-FITC, and blue represents DAPI stained nuclei. **(B)** Intracellular trafficking of internalized milk-exosome/cis-FITC monitored by co-localization with the endosome marker Rab5 (red fluorescence) and lysosome marker LysoTracker Red (red fluorescence) by confocal (red fluorescence microscopy. Green represents cis-FITC, and blue represents DAPI stained nuclei.

To determine the colocalization between endocytic compartment and cis-FITC, A2780CP cells were treated with milk-exosome/cis-FITC, which was followed by co-staining with Rab5 for early endosomes, LysoTracker for lysosomes, respectively. As a control, cis-FITC was incubated with A2780CP cells followed stained with the same organelles, respectively. However, in the group treated with milk-exosome/cis-FITC, cis-FITC had a diffuse cytosolic distribution, not co-localized with Rab5 or LysoTracker (Figure 8B), indicating that cis-FITC evades the endosome trapping. Unlike the group treated with milk-exosome/cis-FITC, in the group A2780CP cells treated with simple cis-FITC, cis-FITC were detected co-localized with Rab5 and LysoTracker, suggesting that cis-FITC trafficked to and trapped in endosomes as well as lysosomes (Figure 8A), resulting in the restriction of the anti-cancer efficacy, consistent with previous observations that the vitality of A2780CP cells treated with simple cis-FITC is better than treated with simple milk-exosome/cis-FITC (Figure 3A).

To further quantify the co-localization of the cis-FITC and endosomes, colocalization analysis of were conducted. The Mander's Colocalization Coefficient (M) and the Pearson Correlation Coefficient were calculated (Supplementary Table S3). As depicted in Figure 8A, cisplatin-FITC showed a high colocalization (M = 0.82) with endosome (with Alexa Fluor 594 red fluorescence), suggesting they were primarily localized in endosomes. Similarly, the cis-FITC showed a high overlap with lysosomes (marked with LysoTracker red fluorescence) (M = 0.71), these observations suggesting the trafficking of cisplatin-FITC and accumulation to the endosomes and lysosomes. In contrast, once cis-FITC transported through milk-exosome/cis-FITC, showed a much lower colocalization with either endosome or lysosome (Figure 8B). These results confirmed the conclusion be proposed above, that milk-exosomes would regulate cisplatin to evade endosome trapping or lysosome trapping.

**Figure 9:**
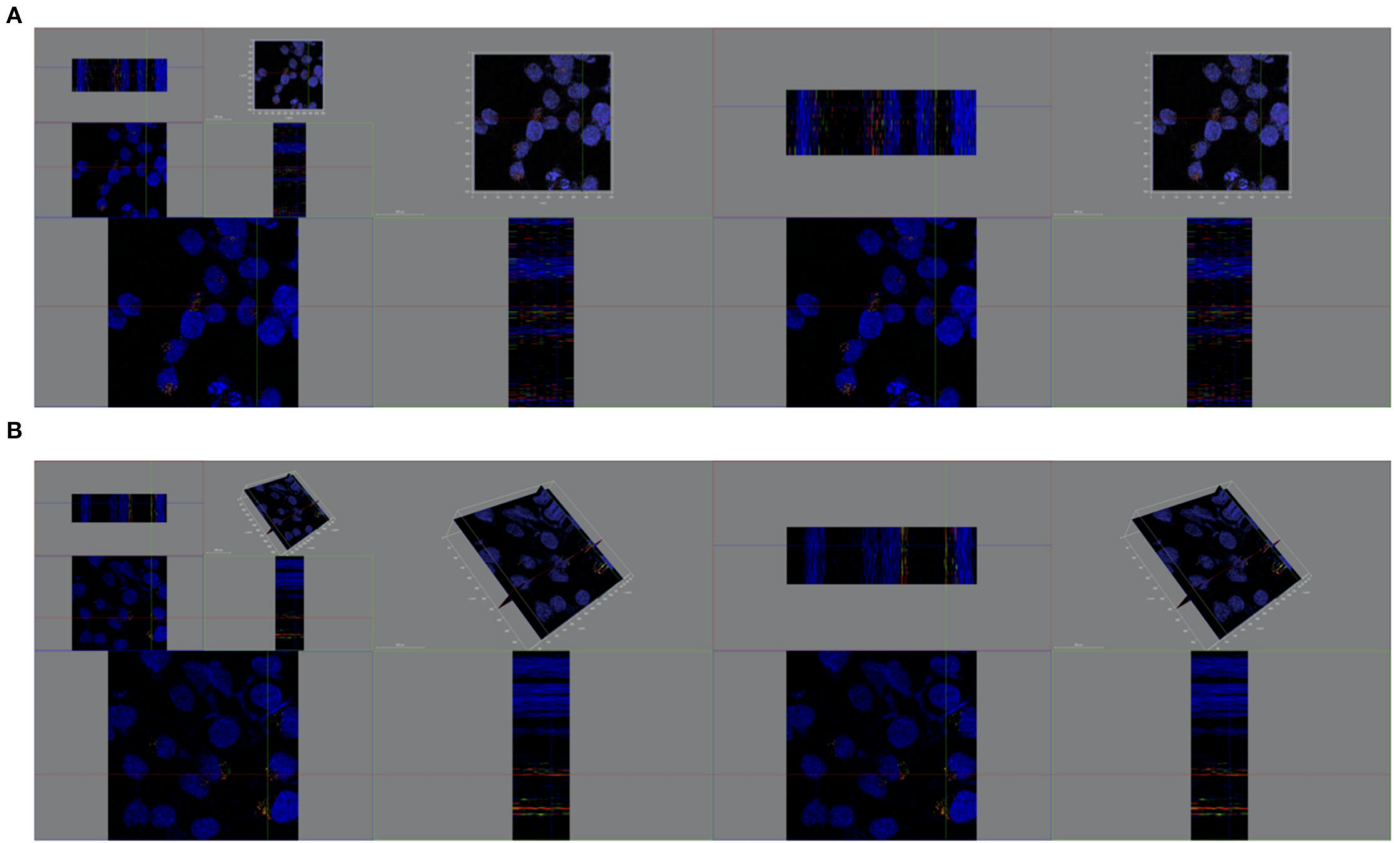
Spatial distribution of cytosolic cisplatin with or without milk-exosomes delivery. **(A)** Spatial distribution of cis-FITC in A2780CP cells visualized from X axis, Y axis, Z axis with milk-exosome delivery. Red represents lysosomes, green represents cis-FITC, and blue represents DAPI stained nuclei. **(B)** Spatial distribution of cis-FITC in A2780CP cells visualized from X axis, Y axis, Z axis without milk-exosome delivery. Red represents endosomes, green represents cis-FITC, and blue represents DAPI stained nuclei.

**Figure 10:**
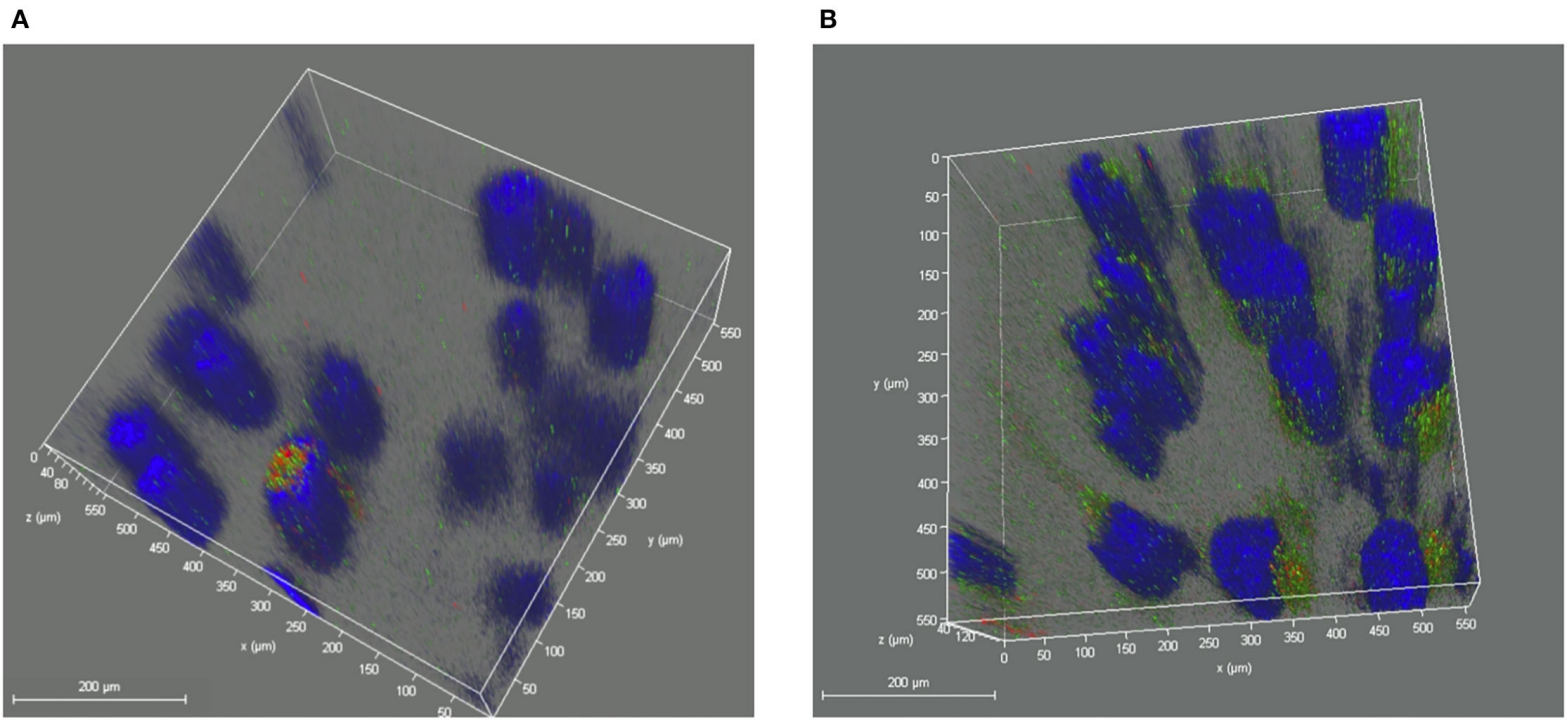
Re-constructed 3D analysis of cisplatin distribution when delivered with or without milk-exosomes. **(A)** Representative re-constructed 3D image of cis-FITC in A2780CP cells with milk-exosome delivery. Red represents endosomes, green represents cis-FITC, and blue represents DAPI stained nuclei. **(B)** Representative re-constructed 3D image of cis-FITC in A2780CP cells with milk-exosome delivery. Red represents endosomes, green represents cis-FITC, and blue represents DAPI stained nuclei. Scale bars are shown to the left.

In the further three-dimensional observation, more diffused cytosol distribution of cisplatin A2780/CP cells was observed in the group treated with milk-exosome/cis-FITC (Supplementary Video S1) when compared with the simple cis-FITC group (Supplementary Video S2). These results from the re-constructed 3D images (Figures 9, 10) are in line with the results from 2D observations.

## Discussion

This study reveals an important mechanism that milk-exosomes enables the loading drug to evade the endosome trapping and enhances the anti-cancer effectiveness of the loading drug in cisplatin-resistant ovarian carcinoma. In this study, exosomes derived from fresh milk could encapsulate the chemotherapeutics cisplatin, deliver the drug into the cisplatin-resistant ovarian cancer, meanwhile enhance the anti-cancer effect of cisplatin *in vitro* and *in vivo*. Further findings show that milk-exosome/cis were internalized into the cisplatin-resistant ovarian cancer *via* the clathrin-independent endocytosis and macropinocytosis, and avoided endosome trapping once enter into the cells, kept the distribution of cisplatin evenly in the cancer cells instead of been trapped or been efflux, enhanced the anti-cancer effectiveness of cisplatin.

Milk exosomes (9) exhibited potential in carrying multiple kinds of small drug molecules. In a long time, exosomes were perceived similar to liposomes, for both of the two kinds of nanocarriers (32) are with bi-lipid membrane structure. However, exosomes (including milk exosomes) are natural biogenesis vesicles (33) consist of multiple kinds of proteins and lipids, which might determine the approach (34–36) enter the cells and subsequent drug delivery ability that different from the traditional artificial nanocarriers. What's more, exosomes isolated from milk may be more compatible with the oral route than intravenous administration, which would be a huge advantage for further clinical applications.

For a long time, cisplatin-based chemotherapeutics are used as first-line therapy against ovarian cancer, unfortunately, cisplatin resistance is still the main unsettled issue. It is widely acknowledged that cisplatin is transported mainly through the plasma membrane transporter called hCtr1 (23, 37), suggesting that the resistant cancer cells which express low level of hCtr1 would limit the amount of cisplatin enter the ovarian cancer cells. Secondly, a large population of cisplatin is trapped in endosomes and lysosomes (22) since they enter resistant ovarian cancer cells, subsequently be pumped out (37) the cells or be degraded during the sequestration. Based on the mechanisms occurs in cisplatin-resistant ovarian carcinoma, the therapeutic efficiency is often comprised due to entrapment in endosomes after endocytosis (36). These obstacles led to the compromised therapeutic effects of cisplatin in ovarian carcinoma once cisplatin-resistance arise.

Based on the results from proteome analysis from milk-exosome, a large proportion of proteins enrich in the endocytosis pathway. To figure out whether the approaches (the cisplatin be transported into cells) play roles in the anti-cancer effect of cisplatin in cisplatin-resistant ovarian cancer, we used several pharmacological molecule inhibitors and found that milk-exosomes deliver cisplatin mainly *via* clathrin-independent endocytosis and macropinocytosis, and then we verified the result through RNAi-mediated inhibition technology to knock-down the key molecules.

It is the first time to report that milk-exosome deliver cisplatin into ovarian cancer cells through clathrin-independent endocytosis and macropinocytosis, rather than rely on direct fusion or well-known plasma membrane transporter hCtr1. What's more, it is the first time to propose that milk-exosome/cisplatin regulate the cisplatin evade the endosome trapping and lysosome trapping after entered the cisplatin-resistant ovarian cancer cells. In addition, the 3D evaluation confirmed the results in another spatial dimensions (Figures 9, 10), which enhanced the credibility of the conclusion.

These founding explain the observation that milk-exosome enhance the anti-cancer effectiveness of cisplatin when compared with the simple cisplatin treatment in cisplatin-resistant ovarian carcinoma. As for whether milk-exosomes could enhance the anti-cancer effectiveness *via* regulating evade endosome trapping in other drug resistant cancer including drug-resistant lung carcinoma, colorectal cancer, cerebral glioma and so on, these meaningful issues need further adequate studies.

In spite of that there might be a little difference among the encapsulation efficacy for load cisplatin into milk-exosomes conducted by different researchers, the loading effectiveness is confirmed in several studies. Also, whether milk-exosomes could encapsulate and deliver other therapeutics efficiently including other chemotherapeutic drugs, microRNAs or some molecules; and the difference of the loading ability of milk-exosomes between diverse other drugs/molecules needs to be evaluated through a large quantity of further studies.

According to the present clinical guideline, several drug delivery systems were applied among patients, including liposomes (38, 39) were used as drug-nanocarriers for treating ovarian cancer or other carcinomas so as to improve the drug delivery efficiency and reduce side effects, albumin-bound paclitaxel (40, 41) was used to increase the stability *via* albumin modification. However, these current strategies still failed to overcome the secondary drug-resistance after the first-line chemotherapy among diverse carcinomas including ovarian cancer, which means that the application of these therapeutics are limited once drug-resistance occurs. In this study, milk-exosome could deliver the encapsulated cisplatin into cisplatin-resistant ovarian cancer cells and enhance the anti-cancer effectiveness when compared with the simple treatment of cisplatin. However, the comparison of anti-cancer effectiveness against drug-resistant cancer between milk-exosome delivery and liposomes delivery in not included in this study, needs to be further investigated.

Considering the sources of species about the milk-exosomes, some concerns about the immune response arose. Based on the observations from this study, there is no significant side effect was observed *in vivo*. In addition, some other previously studies (1, 31) confirmed that exosome is a kind of ideal candidate of drug nanocarrier for its low immunogenicity (29, 42, 43). Some preciously studies (29) focusing on the milk-derived bovine milk reported that there was no evidence indicating milk-exosome would bring to immune response. The limitations of the current study including: (1) lacking the immune response results; (2) lacking of toxicity results of milk-exosome/cis and cisplatin alone *in vivo*; (3) proteomic data of ovarian cell treated with exosome/cis vs. cisplatin in needed in the further research. Further studies are needed to verify the security of milk-exosome for the sake of the widespread application.

Above all, this study proposed a new perspective about the milk-exosomes as a novel drug nanocarrier to inhibit the cisplatin-resistant ovarian carcinoma *via* avoiding endosome trapping. These results might provide new directions for understanding the vesicle trafficking into cancer cells and designing new drug delivery nanoparticles.

In this study, exosome derived from fresh milk was isolated to deliver the cisplatin into cisplatin-resistant ovarian carcinoma, inhibited the cancer *via* enabling cisplatin to avoid endosome trapping. The results suggest that milk-exosomes might be a potential nanocarrier for chemotherapeutic drugs against cisplatin-resistant cancer. In addition, this study validates and enriches the theory of intracellular exosome trafficking.

## Data Availability Statement

The datasets presented in this study can be found in online repositories. The names of the repository/repositories and accession number(s) can be found at: The mass spectrometry proteomics data have been deposited to the ProteomeXchange Consortium (http://proteomecentral.proteomexchange.org) *via* the iProX partner repository (1) with the dataset identifier PXD032682.

## Ethics Statement

The animal study was reviewed and approved by Ethics Committee of Fudan University.

## Author Contributions

GZ: writing-original draft and editing. YG and ZZ: writing-original draft. HZ: writing-review and editing and visualization. WL, BX, FZ, and MZ: writing-review and editing. KH and LW: review and editing. JD: writing-review and editing, supervision, and funding acquisition. All authors contributed to the article and approved the submitted version.

## Funding

The research was supported by Chinese National Nature Sciences Foundation (Grant Numbers 81771524 and 91440107) and Fudan University Tomorrow Star Famous Physicians Cultivation Project and Shanghai Medical Center of Key Programs for Female Reproductive Diseases (2017ZZ01016).

## Conflict of Interest

The authors declare that the research was conducted in the absence of any commercial or financial relationships that could be construed as a potential conflict of interest.

## Publisher's Note

All claims expressed in this article are solely those of the authors and do not necessarily represent those of their affiliated organizations, or those of the publisher, the editors and the reviewers. Any product that may be evaluated in this article, or claim that may be made by its manufacturer, is not guaranteed or endorsed by the publisher.

## Supplementary Material

Supplementary Figure S1 Original images for Figure 2E.

Supplementary Figure S2 Assay *via* Ultra Performance Liquid Chromatography (UPLC) of the encapsulation of cisplatin by milk-exosomes. **(A)** Representative figure of UPLC analysis of cisplatin in milk-exosome/cis. **(B)** Representative figure of UPLC analysis of cisplatin in blank control.

Supplementary Figure S3 Analysis of the influence of hCtr1 inhibitor, unlabelled milk-exosomes and temperature upon inhibiting pkh67 labeled milk-exosome/cis internalization *via* confocal microscopy. **(A)** Pkh67 labeled milk-exosome/cis uptake by A2780CP cells pretreated with hCtr1 inhibitor copper sulfate, unlabelled milk-exosomes and 4°C. **(B)** Quantification of pkh67 labeled milk-exosome/cis (green spots) uptake by A2780CP cells (blue fluorescence DAPI labeled nuclei) pretreated in different circumstances by confocal microscopy. Values represent mean ± S.E.M. ^***^, *P* < 0.001, ^****^, *P* < 0.0001.

Supplementary Figure S4 Original images for Figure 7A.

Supplementary Video S1 Re-constructed 3D analysis of cisplatin distribution when delivered with milk-exosomes/cis. Re-constructed 3D video of cis-FITC distribution in A2780CP cells with milk-exosome/cis delivery. Red represents endosomes, green represents cis-FITC, and blue represents DAPI stained nuclei.

Supplementary Video S2 Re-constructed 3D analysis of cisplatin distribution when delivered without milk-exosomes. Re-constructed 3D video of cis-FITC distribution in A2780CP cells without milk-exosome delivery. Red represents endosomes, green represents cis-FITC, and blue represents DAPI stained nuclei.

Supplementary Table S1 siRNA sequence.

Supplementary Table S2 Characteristics and loading abilities of exosome and exosome/cis.

Supplementary Table S3 Calculated Mander's Colocalization Coefficient (M) and the Pearson Correlation Coefficient in Figure 8.
