# Supplementary figures and images for: Exosome Mediated Cytosolic Cisplatin Delivery Through Clathrin-Independent Endocytosis and Enhanced Anti-cancer Effect via Avoiding Endosome Trapping in Cisplatin-Resistant Ovarian Cancer
Source: Front Med (Lausanne). 2022 May 3;9:810761. doi: 10.3389/fmed.2022.810761 (PMC9113028; doi:10.3389/fmed.2022.810761)

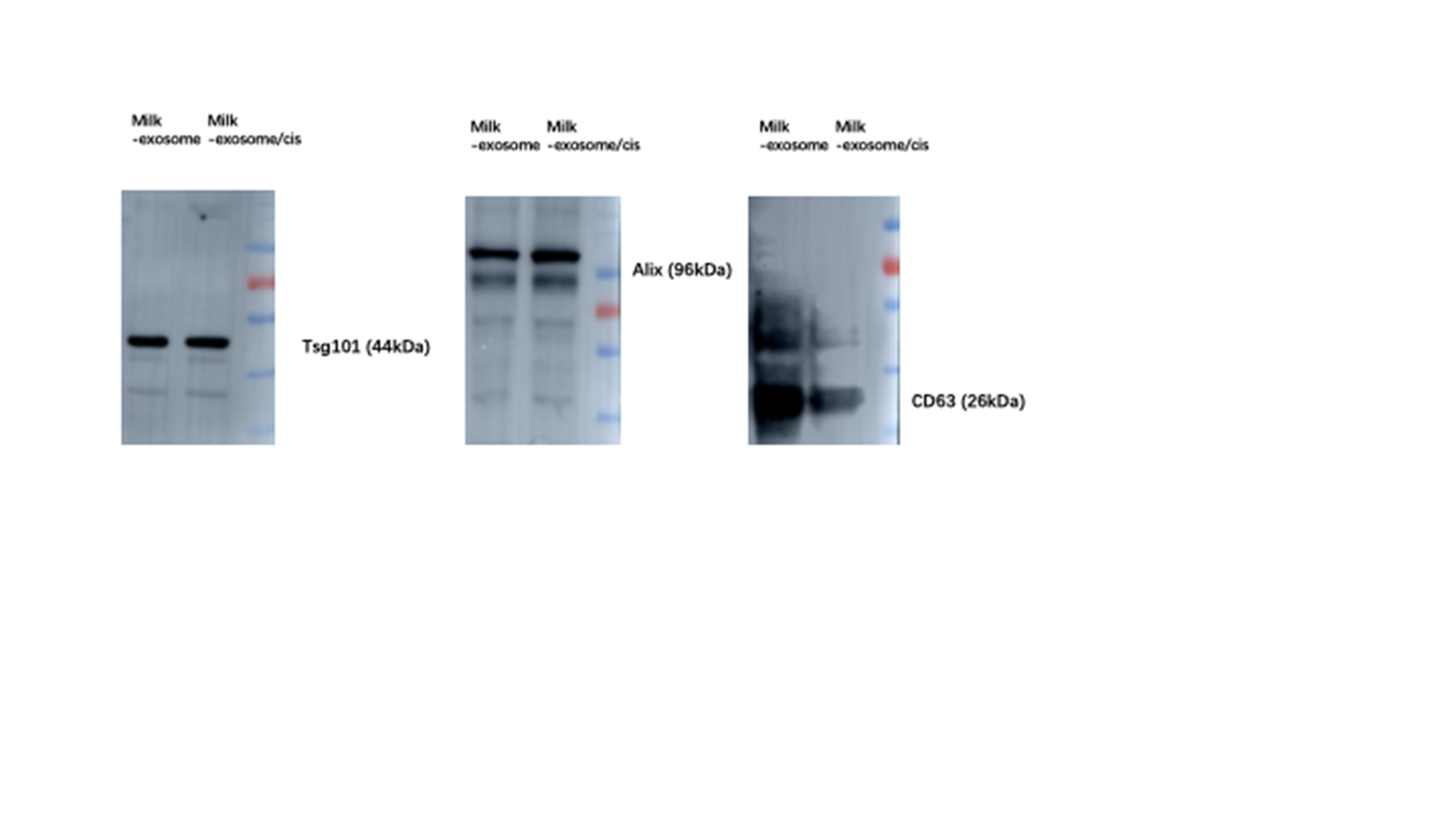

Supplement: Supplementary Figure S1 — Original images for Figure 2E. [file Image_1.TIFF]

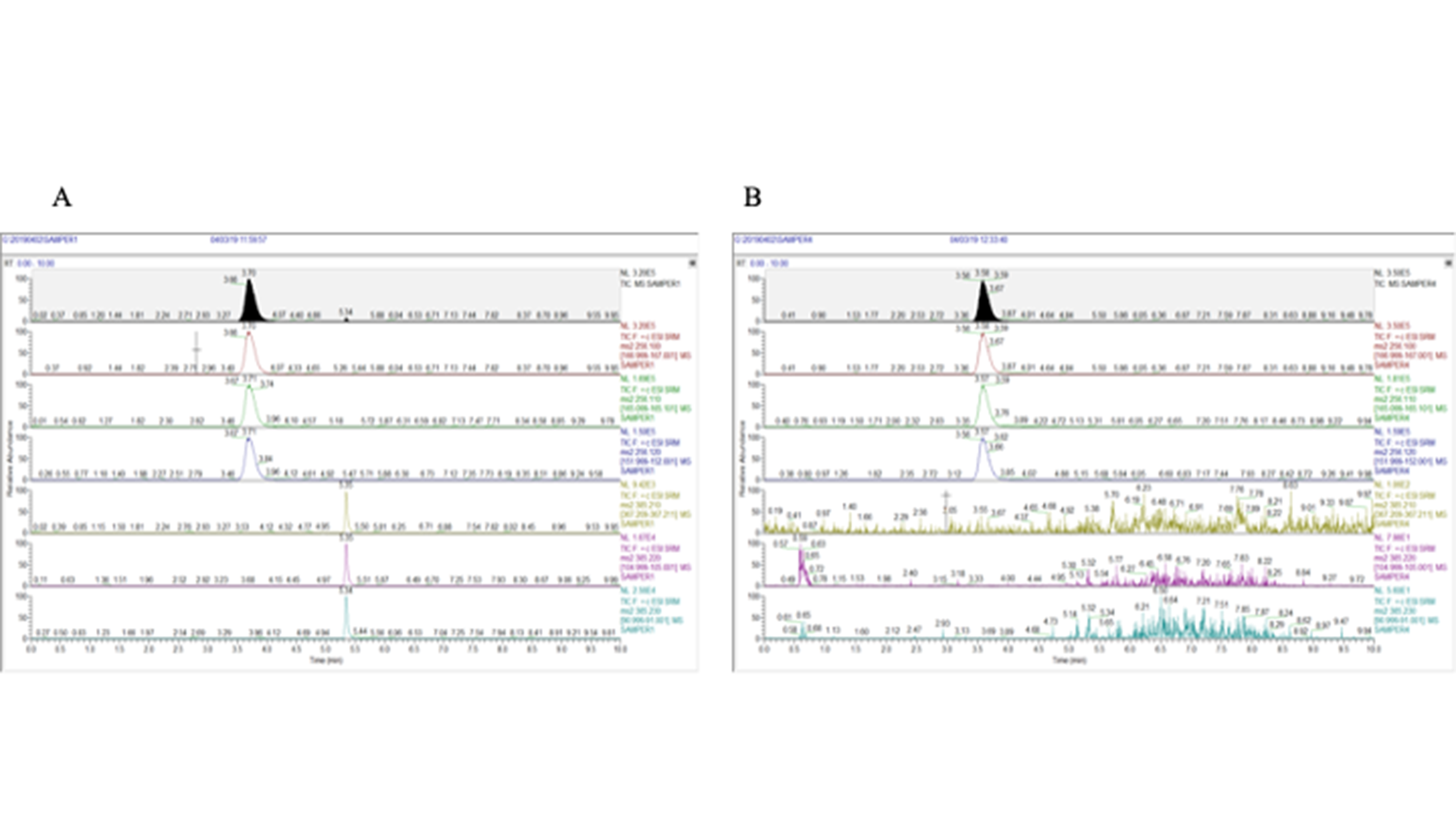

Supplement: Supplementary Figure S2 — Assay via Ultra Performance Liquid Chromatography (UPLC) of the encapsulation of cisplatin by milk-exosomes. (A) Representative figure of UPLC analysis of cisplatin in milk-exosome/cis. (B) Representative figure of UPLC analysis of cisplatin in blank control. [file Image_2.TIFF]

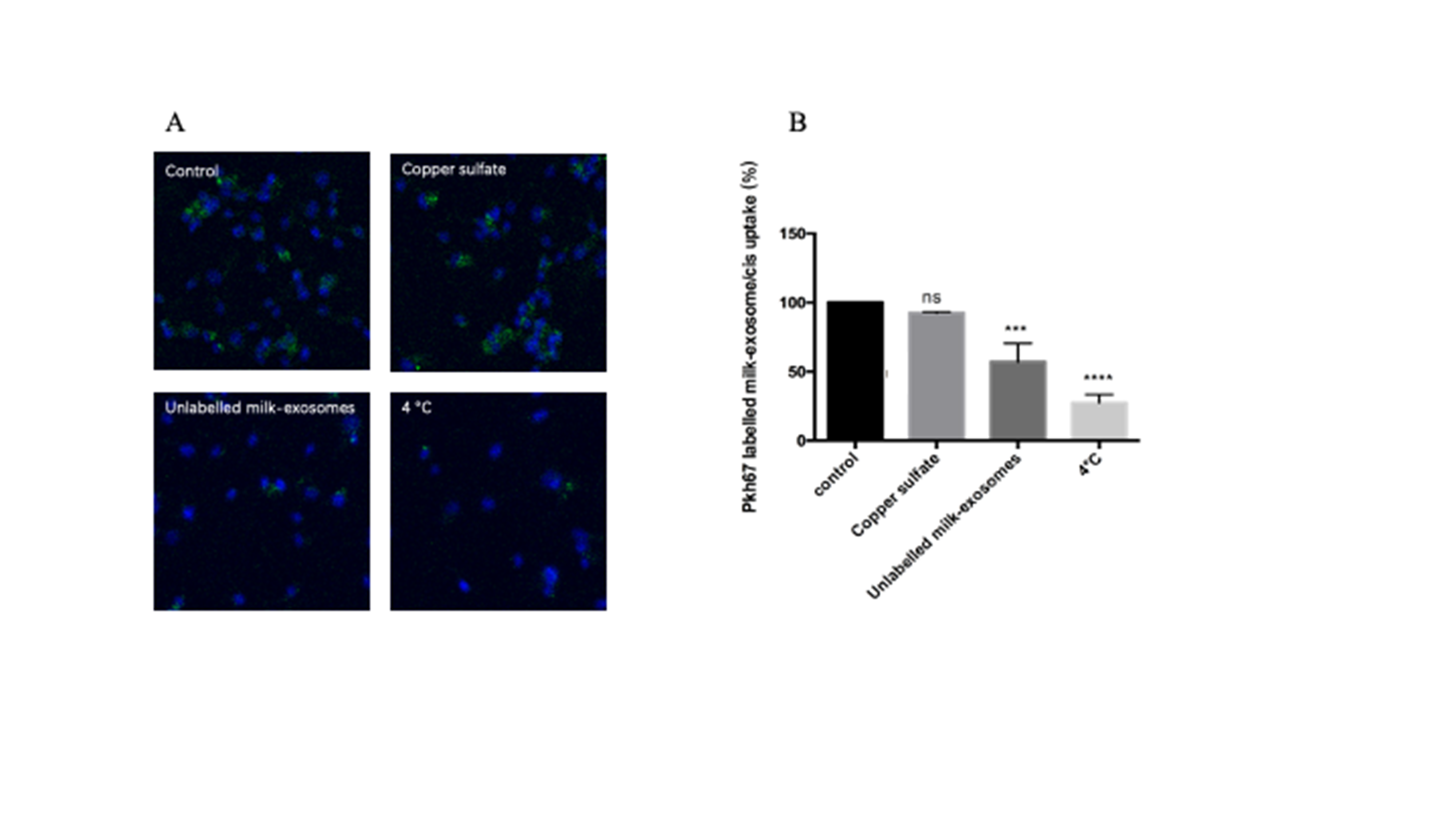

Supplement: Supplementary Figure S3 — Analysis of the influence of hCtr1 inhibitor, unlabelled milk-exosomes and temperature upon inhibiting pkh67 labeled milk-exosome/cis internalization via confocal microscopy. (A) Pkh67 labeled milk-exosome/cis uptake by A2780CP cells pretreated with hCtr1 inhibitor copper sulfate, unlabelled milk-exosomes and 4°C. (B) Quantification of pkh67 labeled milk-exosome/cis (green spots) uptake by A2780CP cells (blue fluorescence DAPI labeled nuclei) pretreated in different circumstances by confocal microscopy. Values represent mean ± S.E.M. ***, P < 0.001, ****, P < 0.0001. [file Image_3.TIFF]

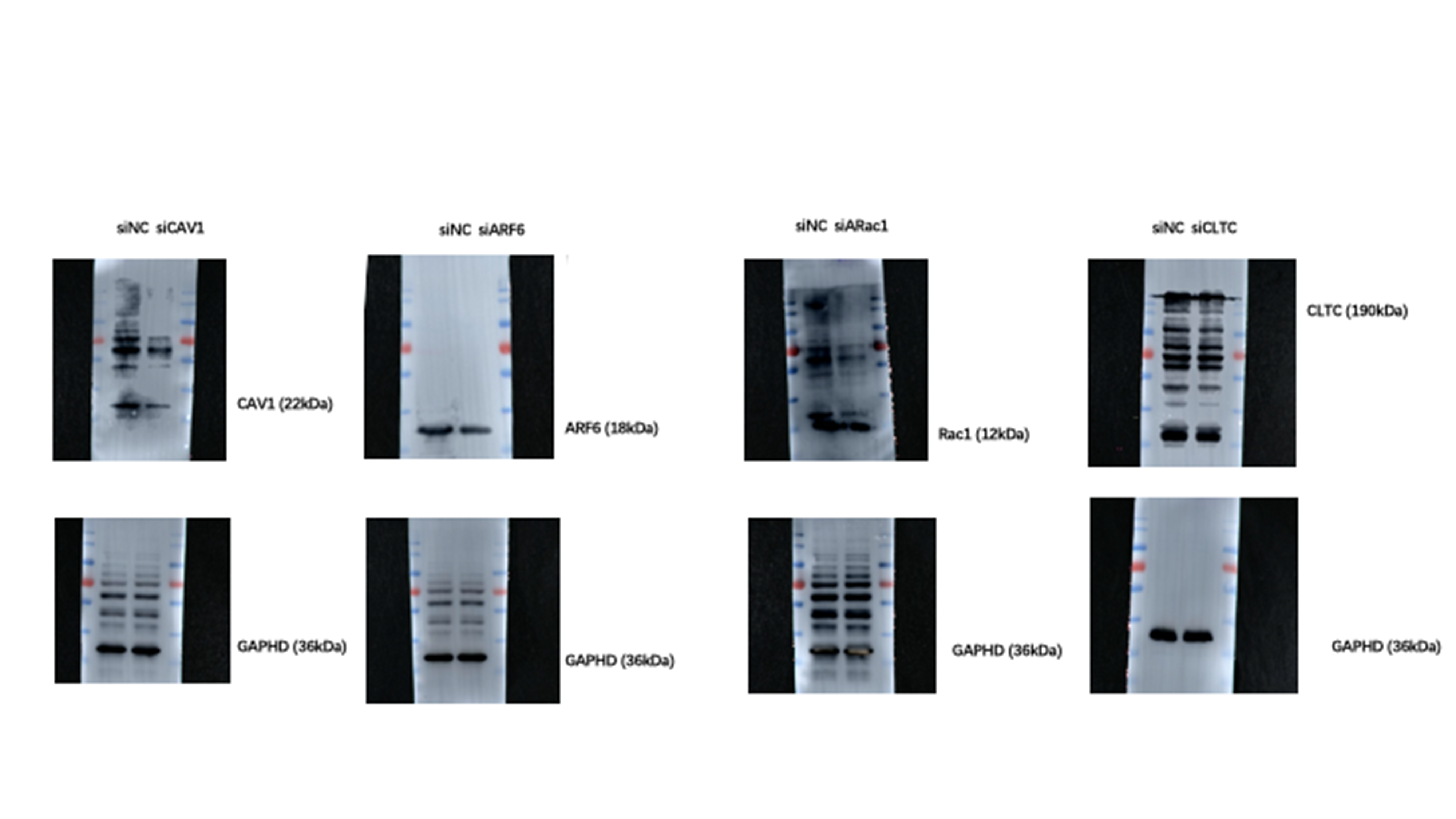

Supplement: Supplementary Figure S4 — Original images for Figure 7A. [file Image_4.TIFF]

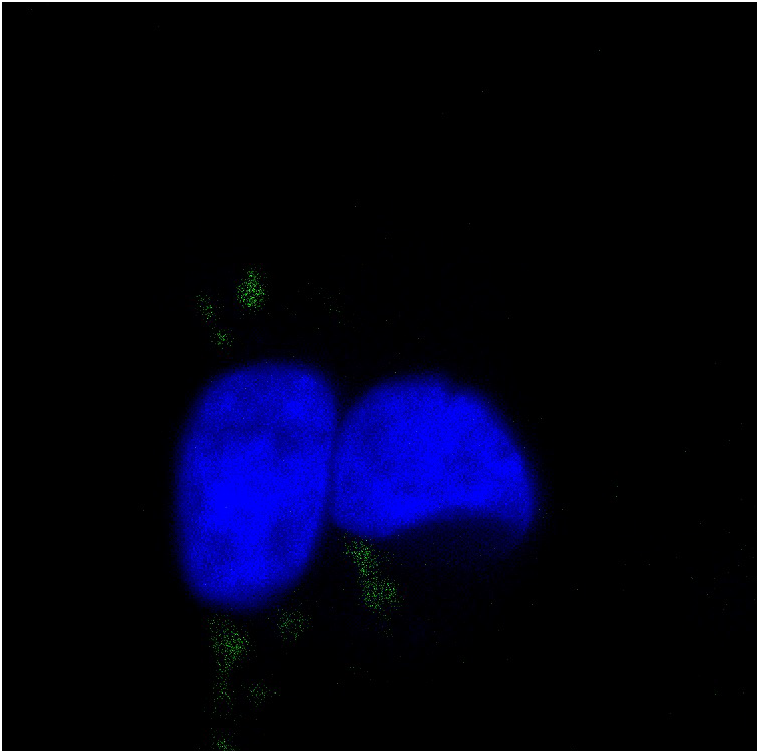

Supplement: Supplementary file 10 [file Data_Sheet_1.ZIP › Source data/original files for microscope images-Figure 7B-siARF6.tiff]

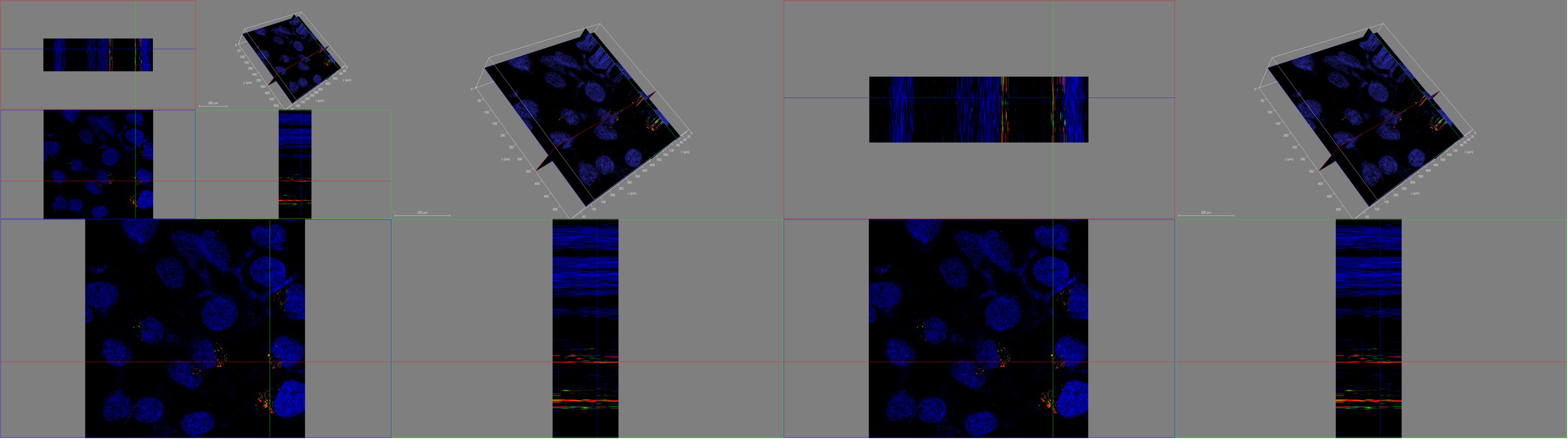

Supplement: Supplementary file 10 [file Data_Sheet_1.ZIP › Source data/original files for microscope images-Figure 9B.tiff]

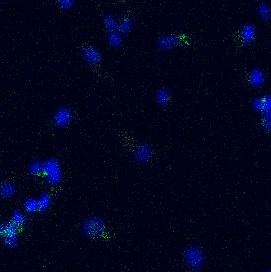

Supplement: Supplementary file 10 [file Data_Sheet_1.ZIP › Source data/original files for microscope images-Figure 6A-Cytochalasin D .tiff]

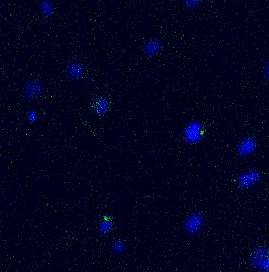

Supplement: Supplementary file 10 [file Data_Sheet_1.ZIP › Source data/original files for microscope images-Figure 6A-EIPA.tiff]

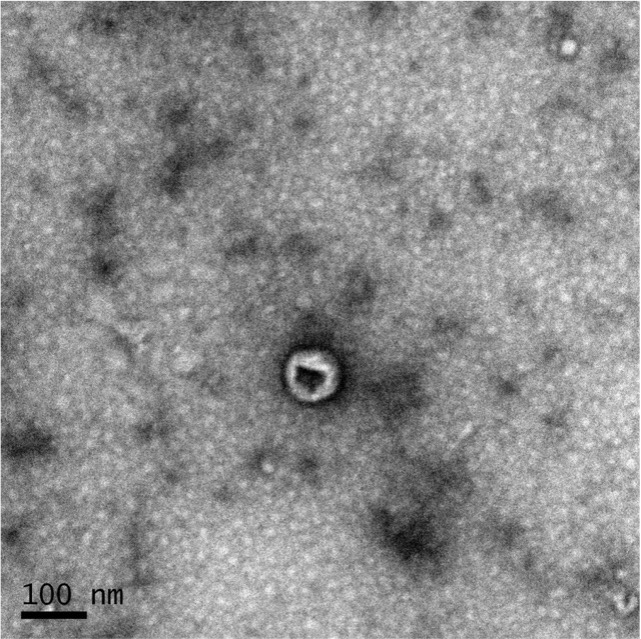

Supplement: Supplementary file 10 [file Data_Sheet_1.ZIP › Source data/original files for transmission electron microscope images-Figure 2A.tiff]

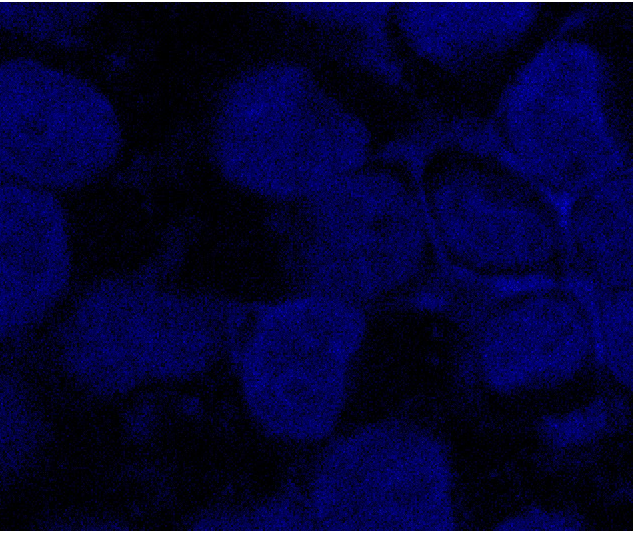

Supplement: Supplementary file 10 [file Data_Sheet_1.ZIP › Source data/original files for microscope images-Figure 8B-DAPI-upper.tiff]

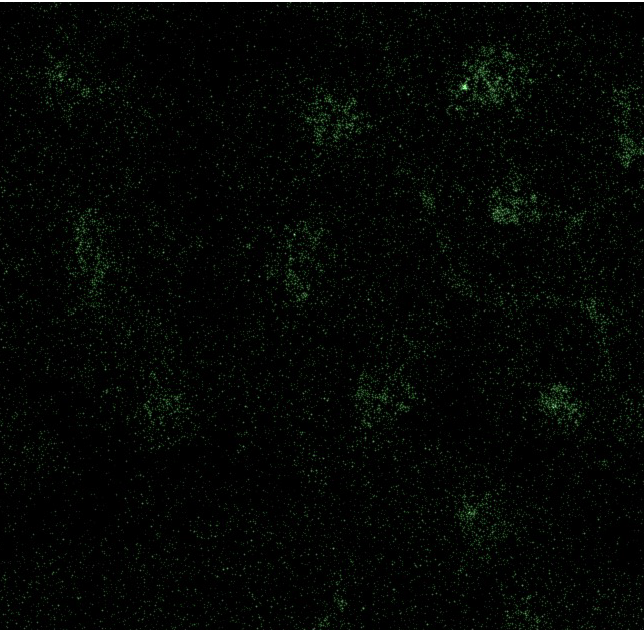

Supplement: Supplementary file 10 [file Data_Sheet_1.ZIP › Source data/original files for microscope images-Figure 8B-Cis-FITC-upper.tiff]

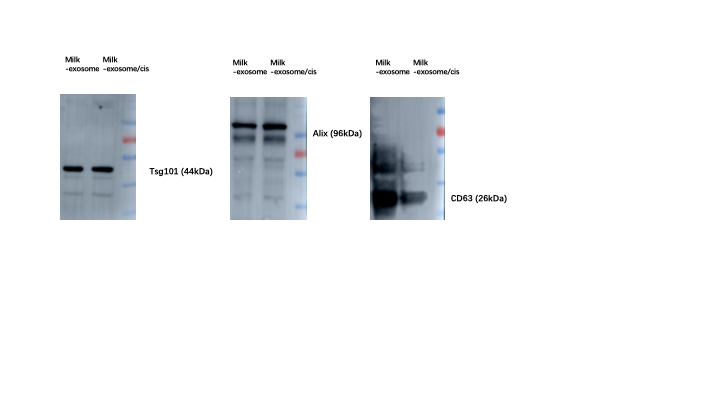

Supplement: Supplementary file 10 [file Data_Sheet_1.ZIP › Source data/Figure S1-original gels-Figure 2E.tiff]

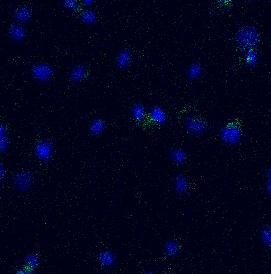

Supplement: Supplementary file 10 [file Data_Sheet_1.ZIP › Source data/original files for microscope images-Figure S3A-Unlabelled milk-exosomes.tiff]

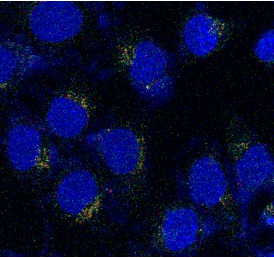

Supplement: Supplementary file 10 [file Data_Sheet_1.ZIP › Source data/original files for microscope images-Figure 8B-Merge-lower.tiff]

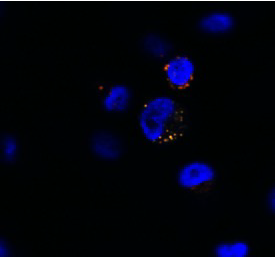

Supplement: Supplementary file 10 [file Data_Sheet_1.ZIP › Source data/original files for microscope images-Figure 8A-Merge-upper.tiff]

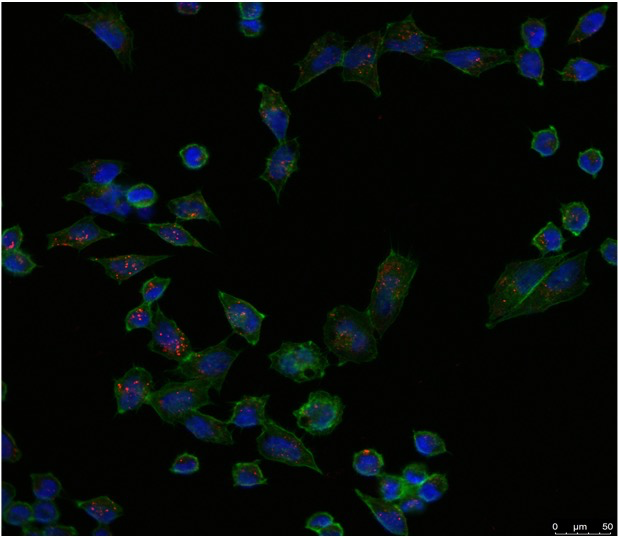

Supplement: Supplementary file 10 [file Data_Sheet_1.ZIP › Source data/original files for transmission electron microscope images-Figure 4A.tiff]

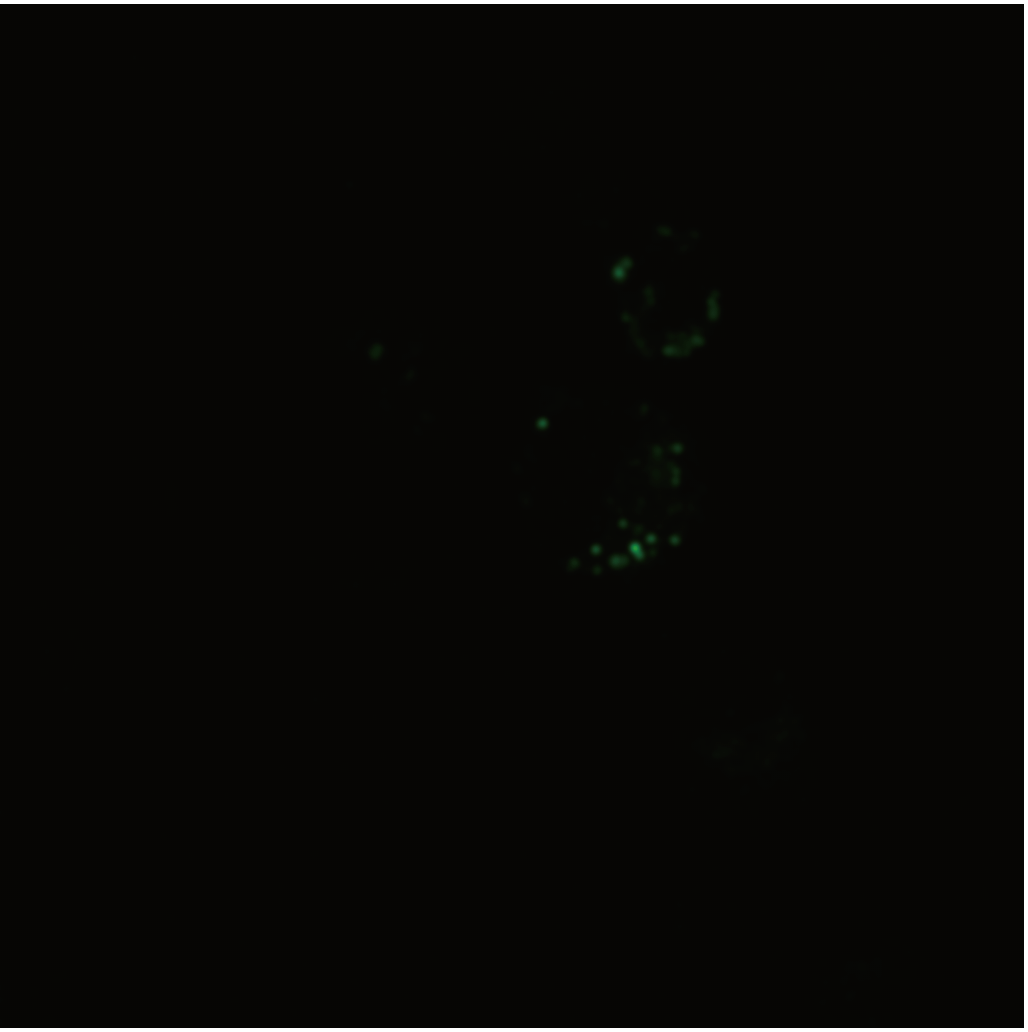

Supplement: Supplementary file 10 [file Data_Sheet_1.ZIP › Source data/original files for microscope images-Figure 8A-Cis-FITC-upper.tiff]

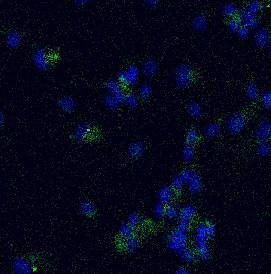

Supplement: Supplementary file 10 [file Data_Sheet_1.ZIP › Source data/original files for microscope images-Figure S3A-Copper sulfate .tiff]

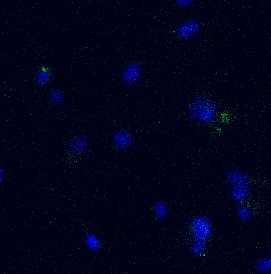

Supplement: Supplementary file 10 [file Data_Sheet_1.ZIP › Source data/original files for microscope images-Figure S3A-4 ┬░C.tiff]

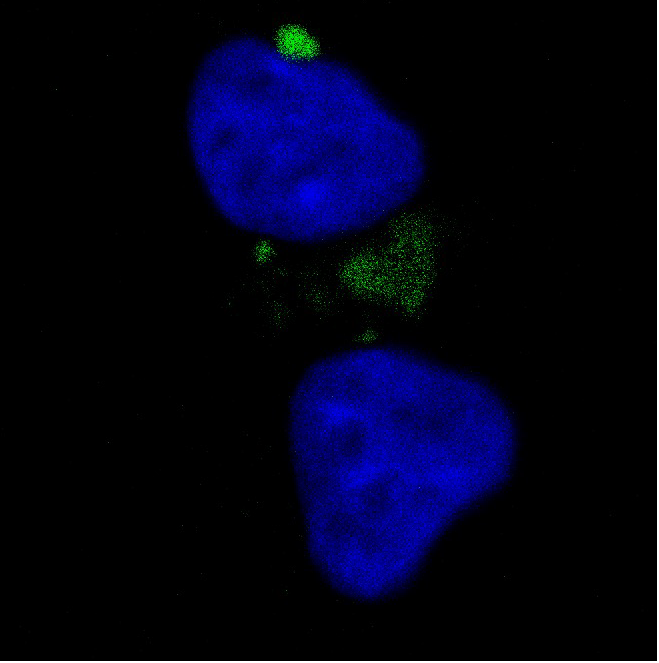

Supplement: Supplementary file 10 [file Data_Sheet_1.ZIP › Source data/original files for microscope images-Figure 7B-siCAV1.tiff]

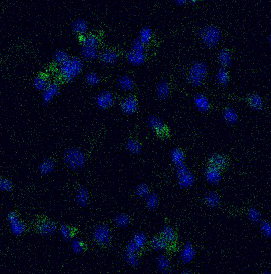

Supplement: Supplementary file 10 [file Data_Sheet_1.ZIP › Source data/original files for microscope images-Figure S3A-Control.tiff]

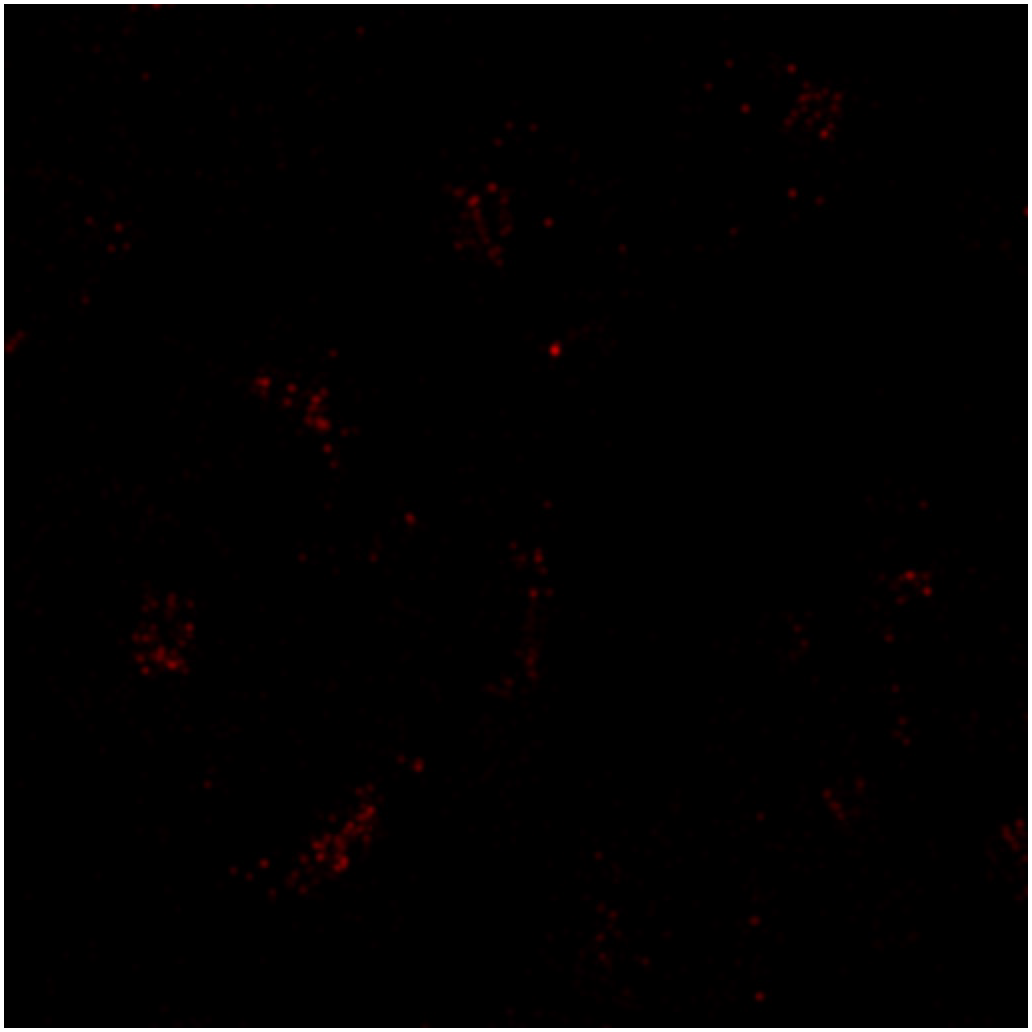

Supplement: Supplementary file 10 [file Data_Sheet_1.ZIP › Source data/original files for microscope images-Figure 8B-Rab5.tiff]

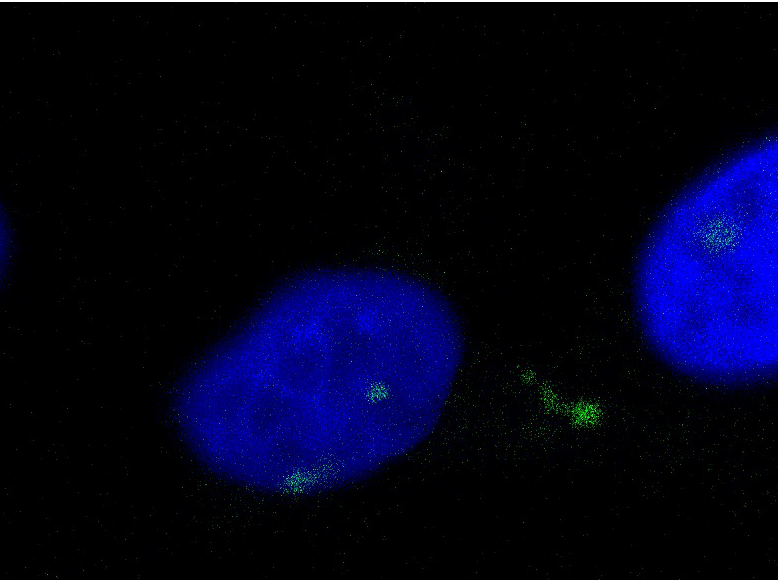

Supplement: Supplementary file 10 [file Data_Sheet_1.ZIP › Source data/original files for microscope images-Figure 7B-siRac1.tiff]

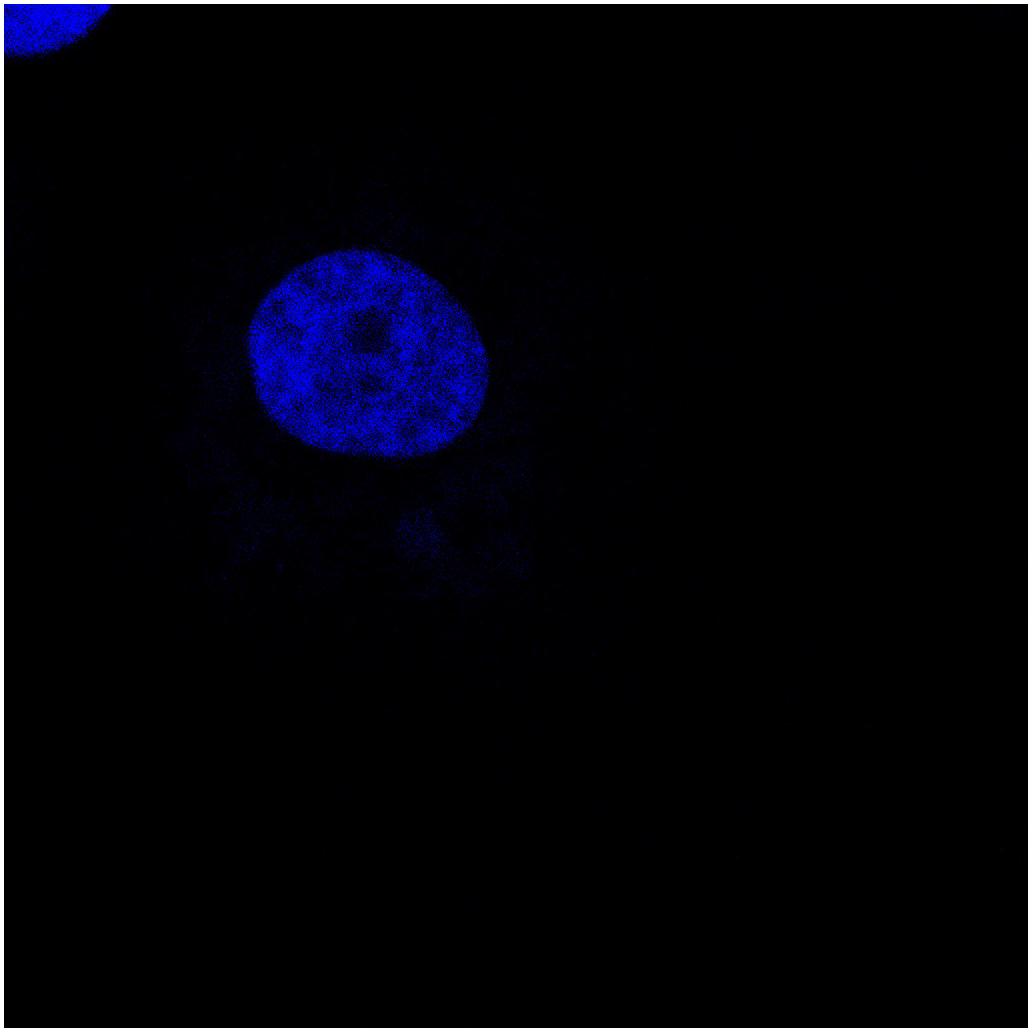

Supplement: Supplementary file 10 [file Data_Sheet_1.ZIP › Source data/original files for microscope images-Figure 8A-DAPI-lower.tiff]

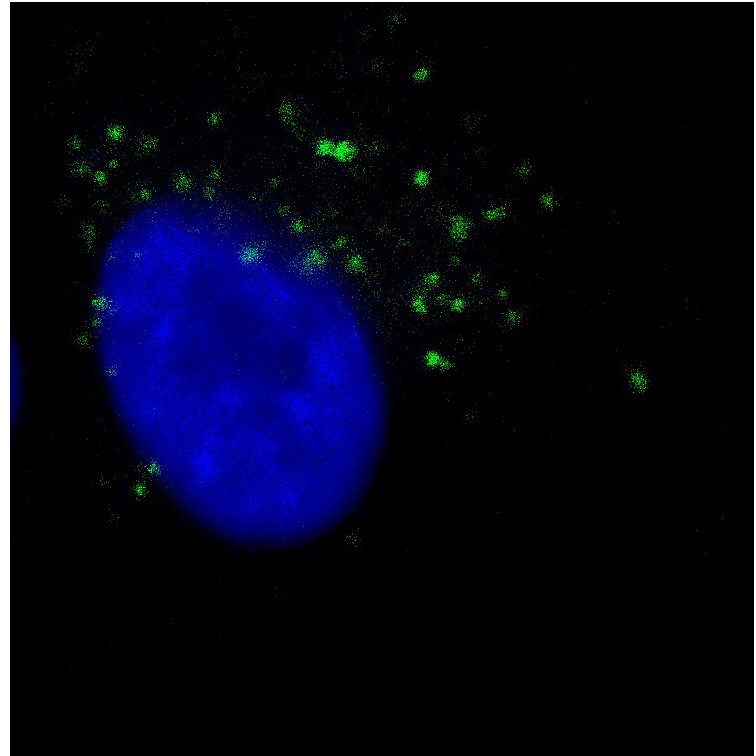

Supplement: Supplementary file 10 [file Data_Sheet_1.ZIP › Source data/original files for microscope images-Figure 7B-siCLTC.tiff]

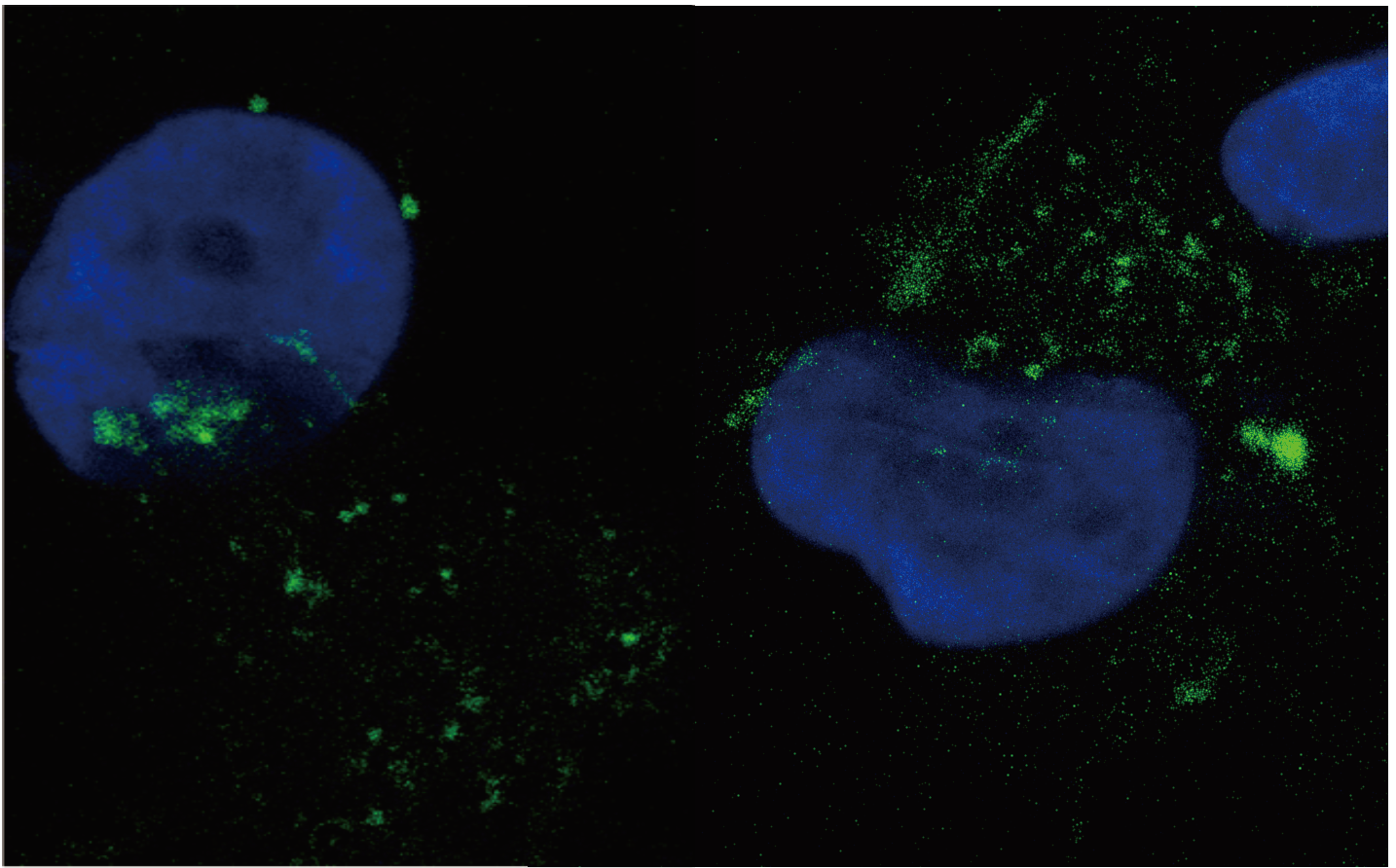

Supplement: Supplementary file 10 [file Data_Sheet_1.ZIP › Source data/original files for microscope images-Figure 7B-Control.tiff]

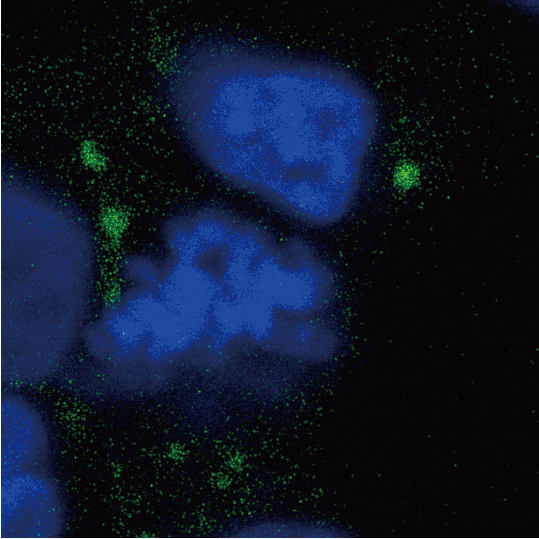

Supplement: Supplementary file 10 [file Data_Sheet_1.ZIP › Source data/original files for microscope images-Figure 7B-siNC.tiff]

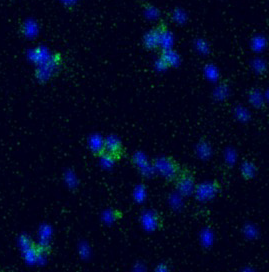

Supplement: Supplementary file 10 [file Data_Sheet_1.ZIP › Source data/original files for microscope images-Figure 6A-Chlorpromazine.tiff]

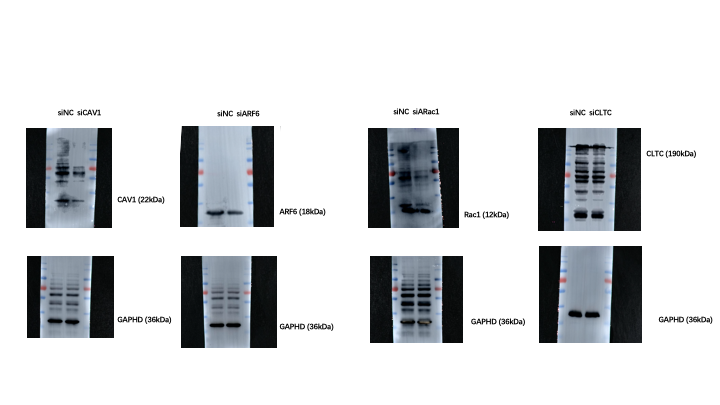

Supplement: Supplementary file 10 [file Data_Sheet_1.ZIP › Source data/Figure S4-original gels-Figure 7A.tiff]

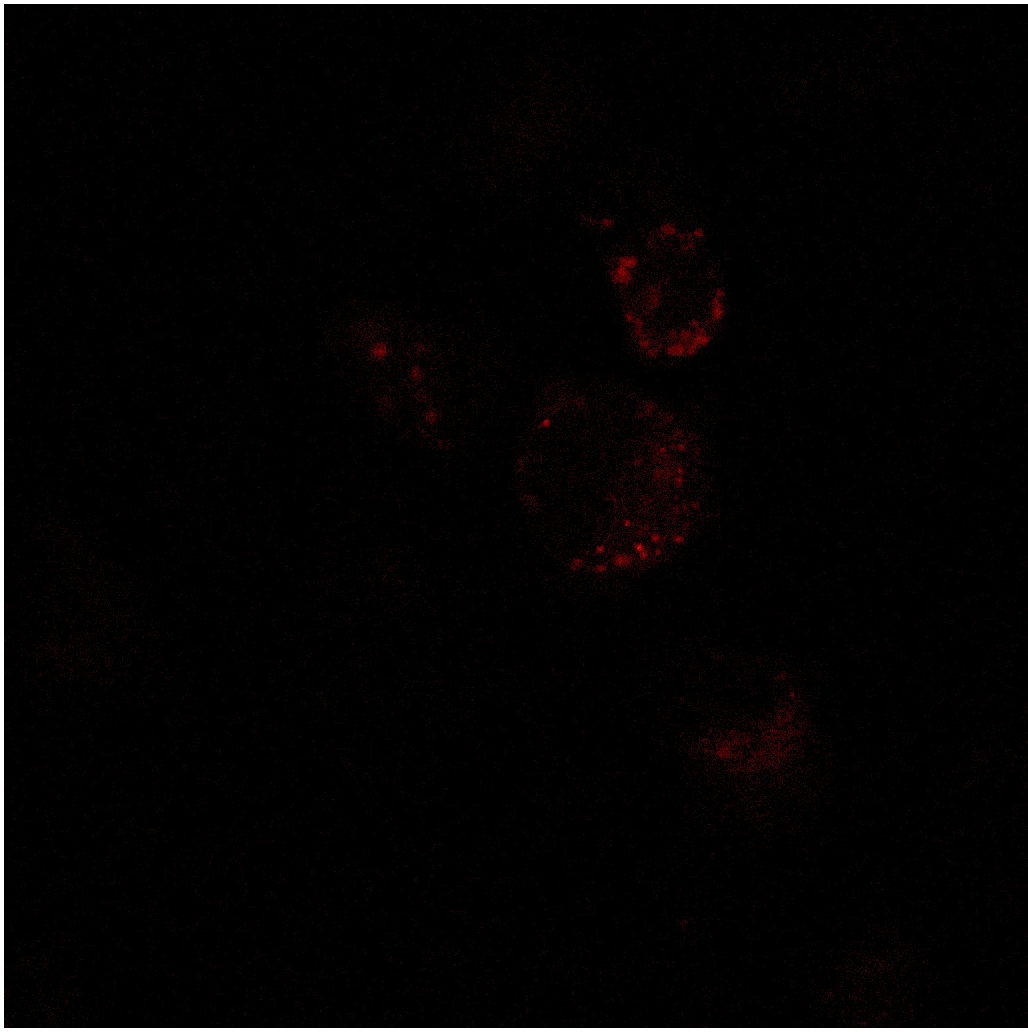

Supplement: Supplementary file 10 [file Data_Sheet_1.ZIP › Source data/original files for microscope images-Figure 8A-Rab5.tiff]

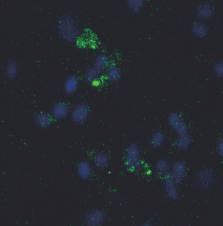

Supplement: Supplementary file 10 [file Data_Sheet_1.ZIP › Source data/original files for microscope images-Figure 6A-Control.tiff]

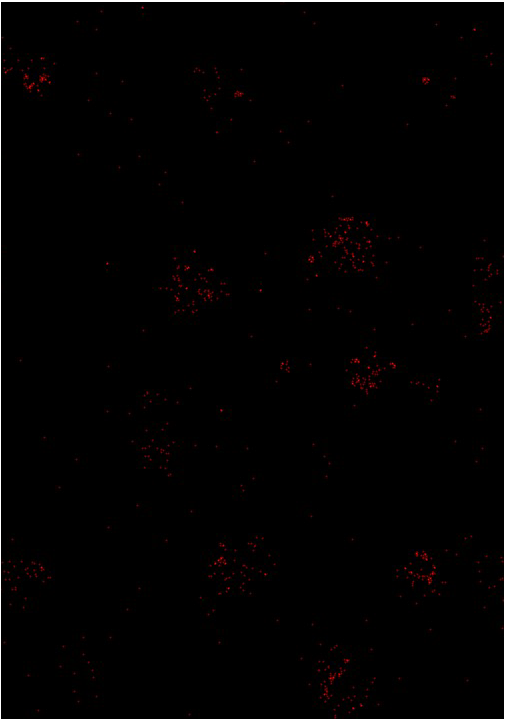

Supplement: Supplementary file 10 [file Data_Sheet_1.ZIP › Source data/original files for microscope images-Figure 8B-Lyso Tracker.tiff]

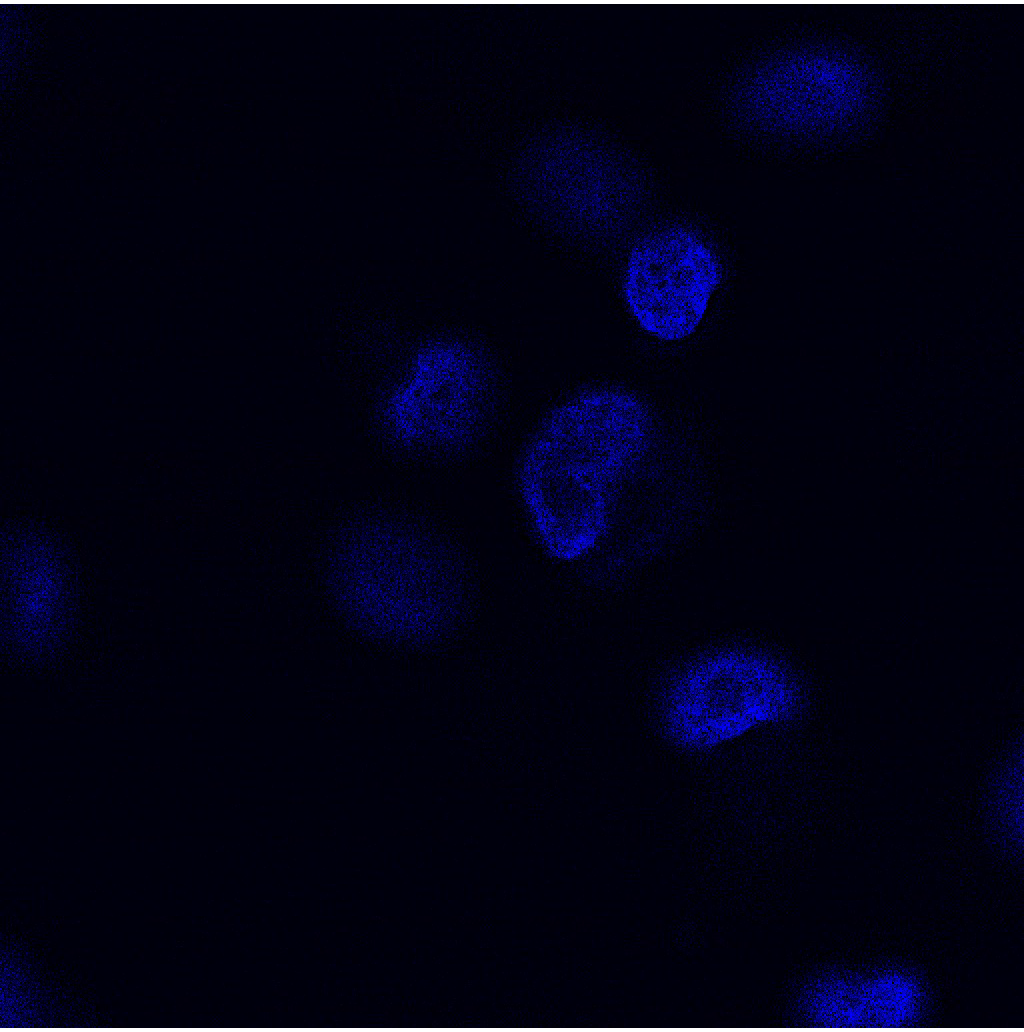

Supplement: Supplementary file 10 [file Data_Sheet_1.ZIP › Source data/original files for microscope images-Figure 8A-DAPI-upper.tiff]

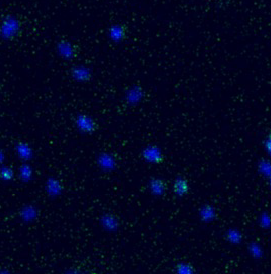

Supplement: Supplementary file 10 [file Data_Sheet_1.ZIP › Source data/original files for microscope images-Figure 6A-Simvastatin.tiff]

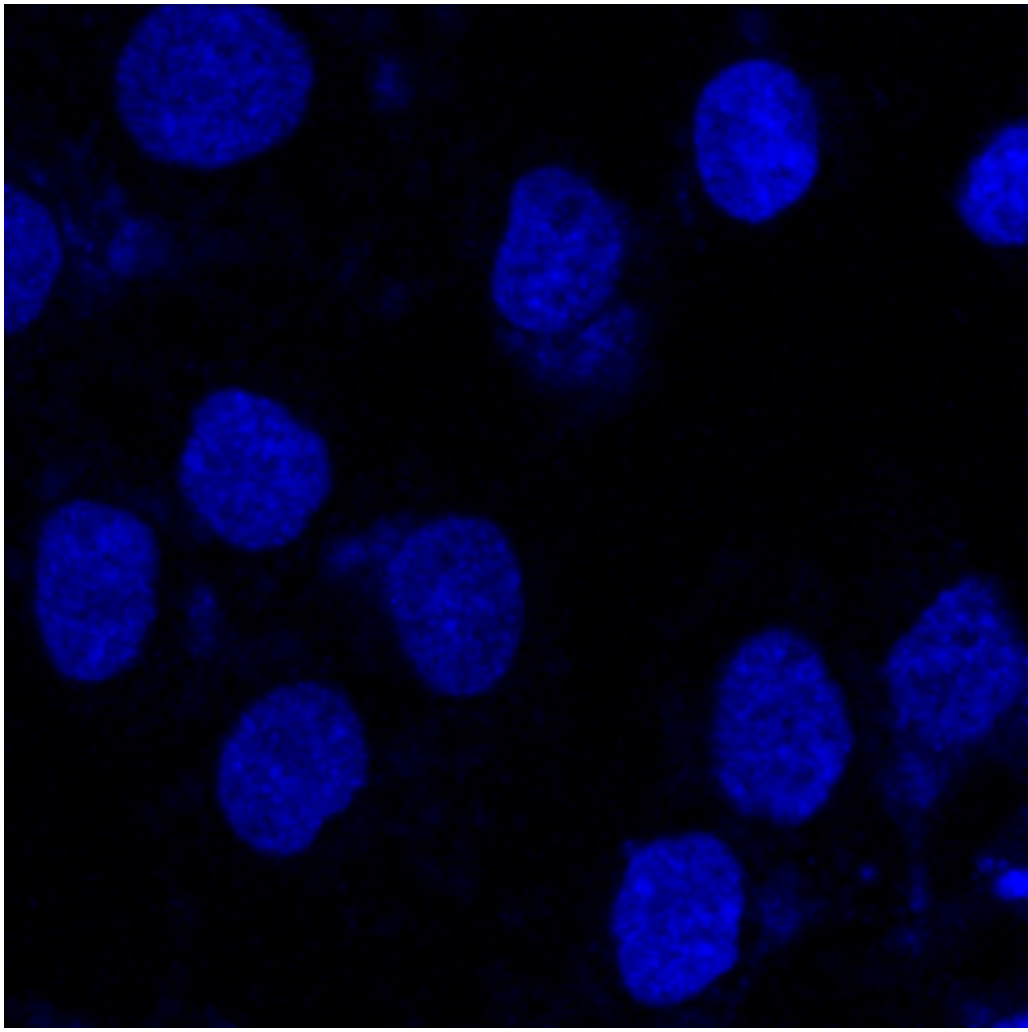

Supplement: Supplementary file 10 [file Data_Sheet_1.ZIP › Source data/original files for microscope images-Figure 8B-DAPI-lower.tiff]

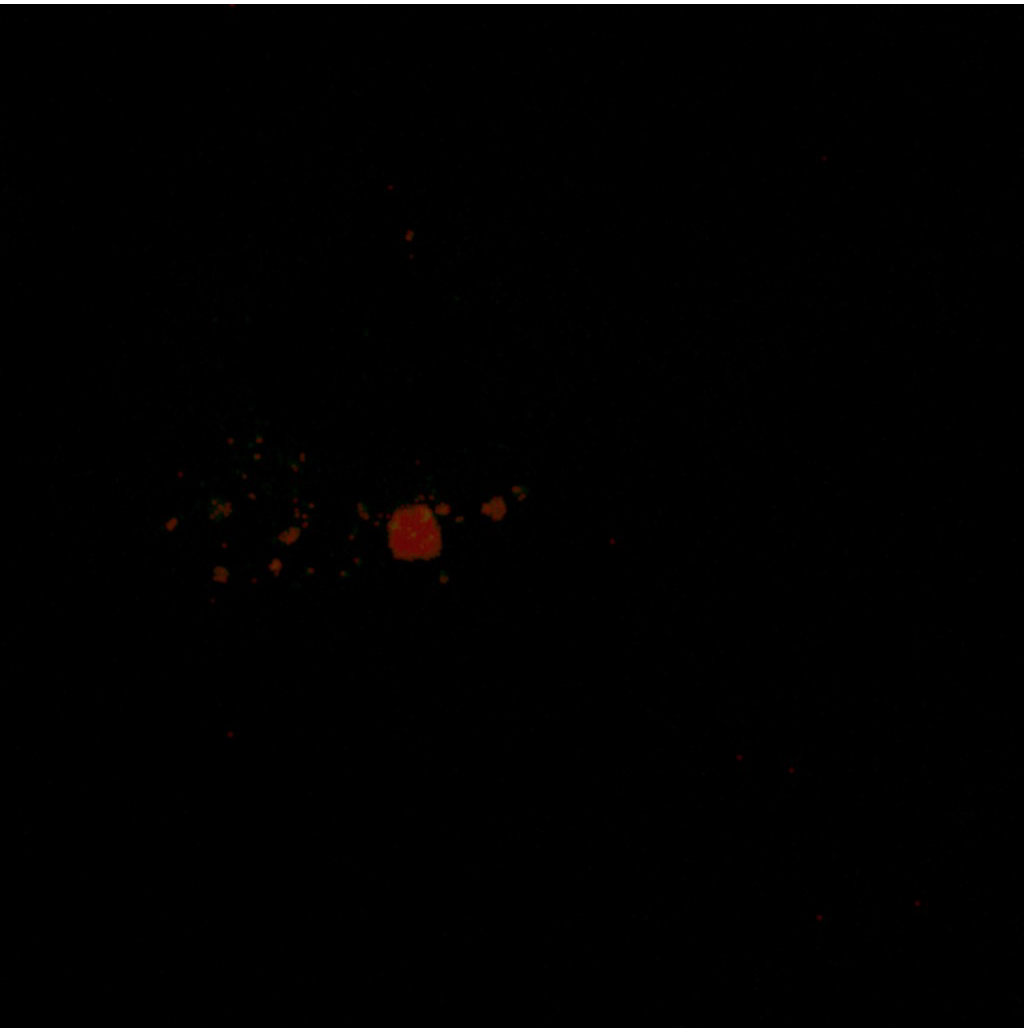

Supplement: Supplementary file 10 [file Data_Sheet_1.ZIP › Source data/original files for microscope images-Figure 8A-Lyso Tracker.tiff]

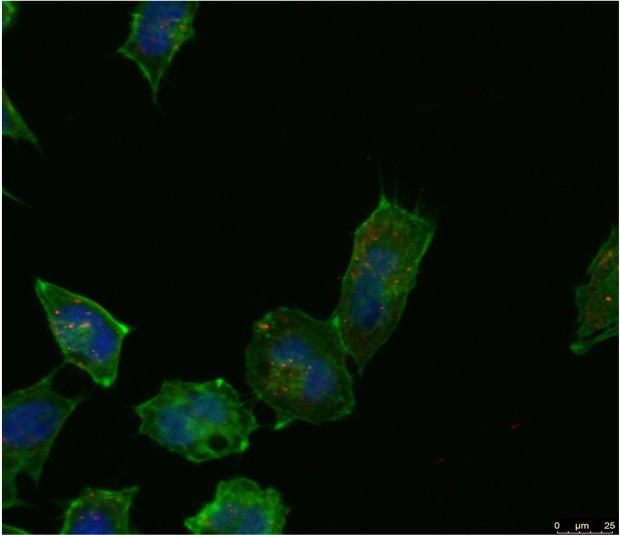

Supplement: Supplementary file 10 [file Data_Sheet_1.ZIP › Source data/original files for transmission electron microscope images-Figure 4B.tiff]

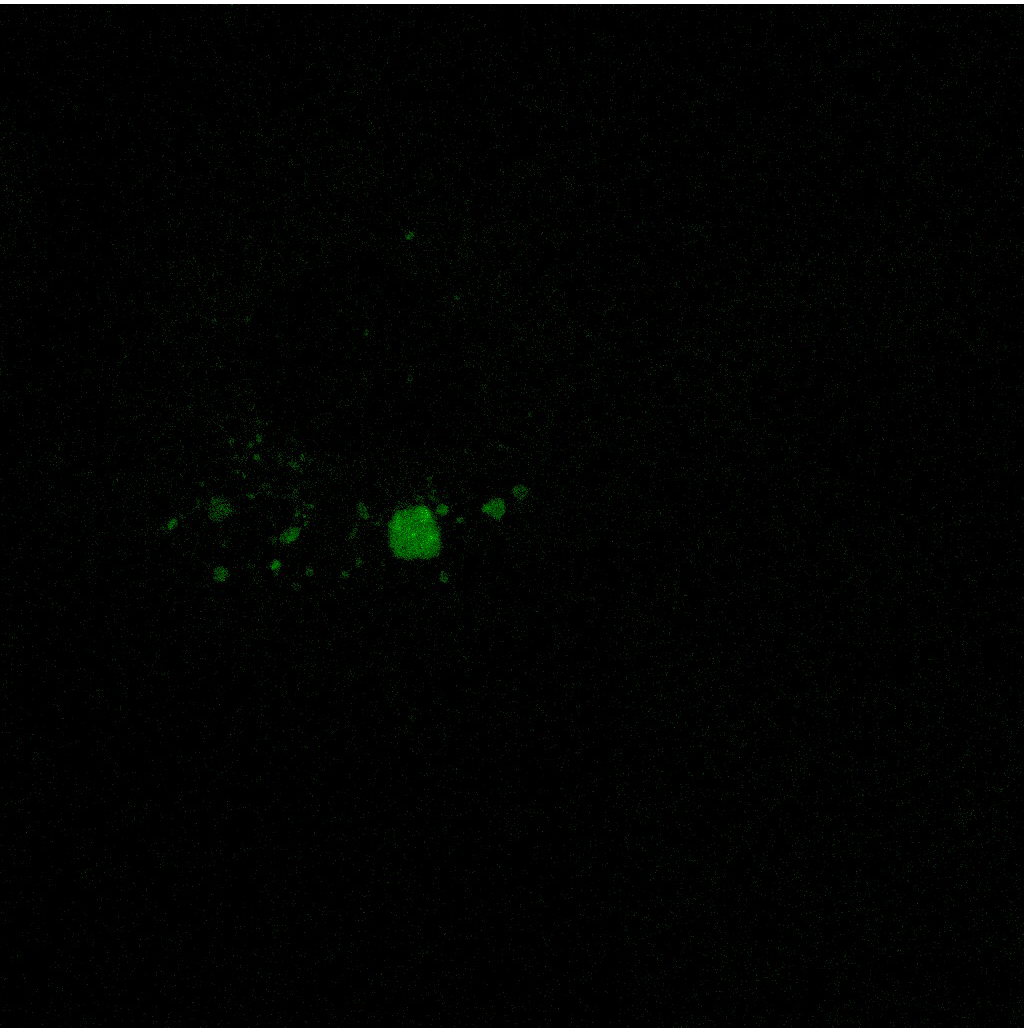

Supplement: Supplementary file 10 [file Data_Sheet_1.ZIP › Source data/original files for microscope images-Figure 8A-Cis-FITC-lower.tiff]

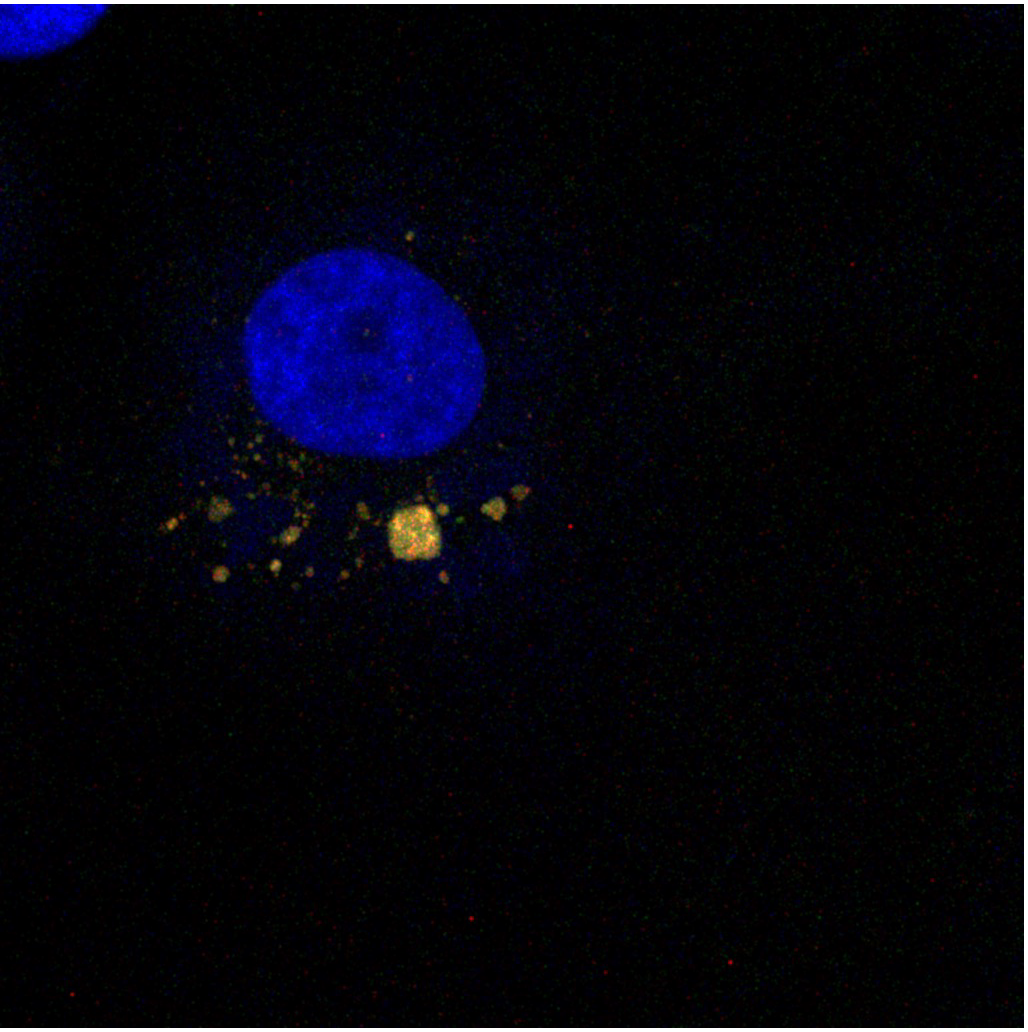

Supplement: Supplementary file 10 [file Data_Sheet_1.ZIP › Source data/original files for microscope images-Figure 8A-Merge-lower.tiff]

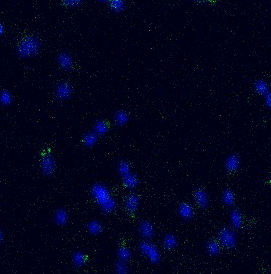

Supplement: Supplementary file 10 [file Data_Sheet_1.ZIP › Source data/original files for microscope images-Figure 6A-Genistein.tiff]

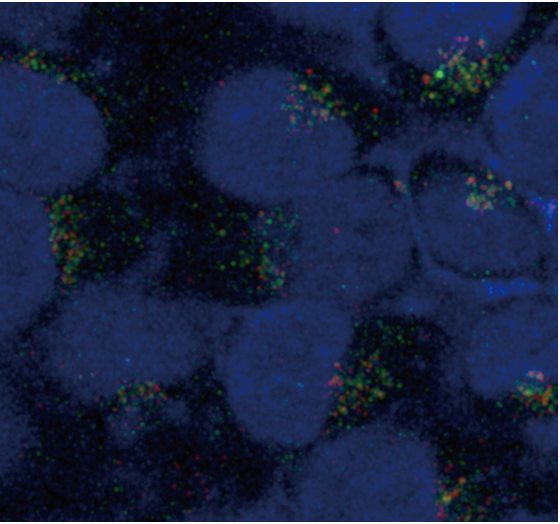

Supplement: Supplementary file 10 [file Data_Sheet_1.ZIP › Source data/original files for microscope images-Figure 8B-Merge-upper.tiff]

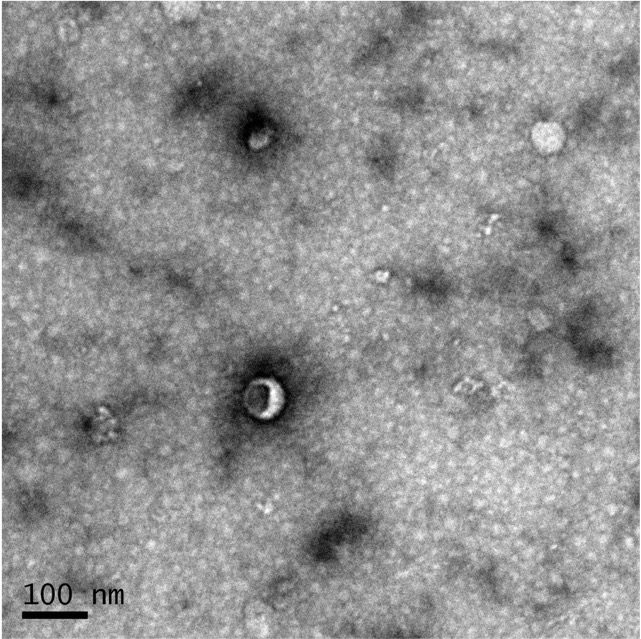

Supplement: Supplementary file 10 [file Data_Sheet_1.ZIP › Source data/original files for transmission electron microscope images-Figure 2B.tiff]

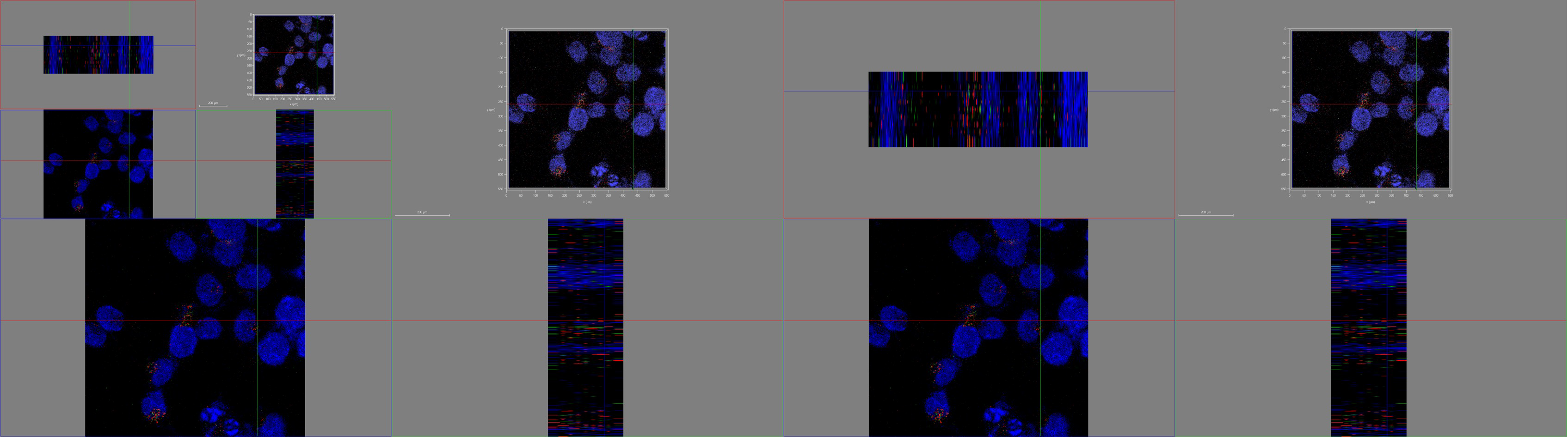

Supplement: Supplementary file 10 [file Data_Sheet_1.ZIP › Source data/original files for microscope images-Figure 9A.tiff]

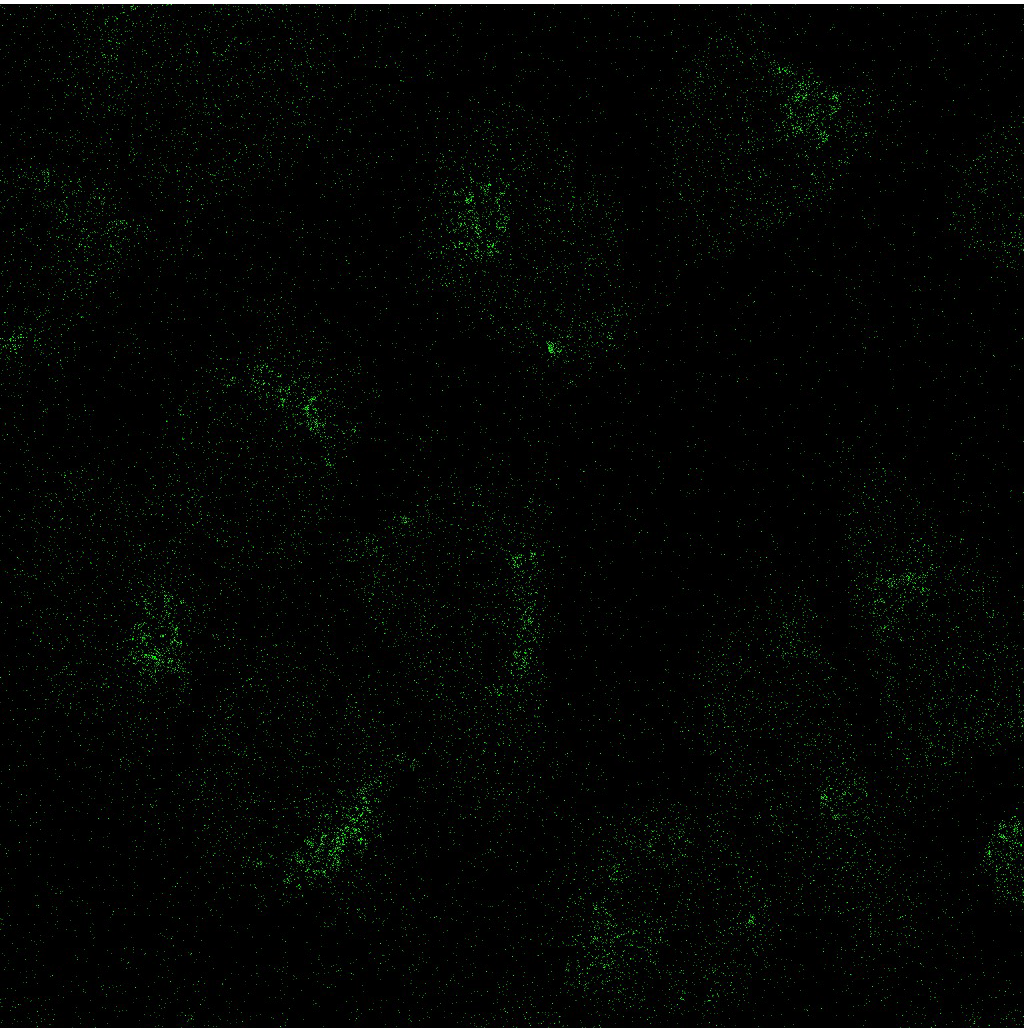

Supplement: Supplementary file 10 [file Data_Sheet_1.ZIP › Source data/original files for microscope images-Figure 8B-Cis-FITC-lower.tiff]
