# Supplementary material for: Exosome Mediated Cytosolic Cisplatin Delivery Through Clathrin-Independent Endocytosis and Enhanced Anti-cancer Effect via Avoiding Endosome Trapping in Cisplatin-Resistant Ovarian Cancer
Source: Front Med (Lausanne). 2022 May 3;9:810761. doi: 10.3389/fmed.2022.810761 (PMC9113028; doi:10.3389/fmed.2022.810761)
Supplement: Supplementary Table S1 — siRNA sequence. [file Table_1.DOCX]

**Table S1. siRNA sequence**

| CAV1 | hsc.rnai.n001172895.12.1 | Sense | 5′-CCUUCACUGUGACGAAAUACUGGtt-3′ |
| --- | --- | --- | --- |
|  |  | Antisense | 5′-AACCAGUAUUUCGUCACAGUGAAGGUG-3′ |
| ARF6 | hsc.rnai.n001663.12.2 | Sense | 5′-CCUCUAACUACAAAUCUUAAUGAgc-3′ |
|  |  | Antisense | 5′-GCUCAUUAAGAUUUGUAGUUAGAGGUU-3′ |
| Rac1 | hsc.rnai.n018890.12.1 | Sense | 5′-GGAACUAAACUUGAUCUUAGGGAtg-3′ |
|  |  | Antisense | 5′-CAUCCCUAAGAUCAAGUUUAGUUCCCA-3′ |
| CLTC | hsc.rnai.n004859.12.7 | Sense | 5′-GCCUUUACAAGGAUGCAAUGCAGta-3′ |
|  |  | Antisense | 5′-UACUGCAUUGCAUCCUUGUAAAGGCUG-3′ |
