# Supplementary material for: Exosome Mediated Cytosolic Cisplatin Delivery Through Clathrin-Independent Endocytosis and Enhanced Anti-cancer Effect via Avoiding Endosome Trapping in Cisplatin-Resistant Ovarian Cancer
Source: Front Med (Lausanne). 2022 May 3;9:810761. doi: 10.3389/fmed.2022.810761 (PMC9113028; doi:10.3389/fmed.2022.810761)
Supplement: Supplementary Table S2 — Characteristics and loading abilities of exosome and exosome/cis. [file Table_2.docx]

**Table S2. Characteristics and loading abilities of exosome and exosome/cis.**

| Formulation | Size (nm) | Cisplatin in milk-exosome/cis (ng) | Drug loading (%) |
| --- | --- | --- | --- |
| Exosome | 73.0 ± 2.1 | 0 | - |
| Exosome/cis | 93.0 ± 0.5 | 1818 | 18 ± 1.0 |
