# Supplementary material for: Exosome Mediated Cytosolic Cisplatin Delivery Through Clathrin-Independent Endocytosis and Enhanced Anti-cancer Effect via Avoiding Endosome Trapping in Cisplatin-Resistant Ovarian Cancer
Source: Front Med (Lausanne). 2022 May 3;9:810761. doi: 10.3389/fmed.2022.810761 (PMC9113028; doi:10.3389/fmed.2022.810761)
Supplement: Supplementary Table S3 — Calculated Mander's Colocalization Coefficient (M) and the Pearson Correlation Coefficient in Figure 8. [file Table_3.docx]

**Table S3. Calculated Mander’s Colocalization Coefficient (M) and the Pearson Correlation Coefficient in Fig. 8.**

|  | With endosome | | With lysosome | |
| --- | --- | --- | --- | --- |
|  | M | P | M | P |
| Cis-FITC | 0.82 | 0.21 | 0.71 | 0.41 |
| Exosome/cis-FITC | 0.11 | 0.02 | 0.06 | 0.03 |
